# Plasma Aldo-Keto Reductase Family 1 Member B10 as a Biomarker Performs Well in the Diagnosis of Nonalcoholic Steatohepatitis and Fibrosis

**DOI:** 10.3390/ijms23095035

**Published:** 2022-05-01

**Authors:** Aron Park, Seung Joon Choi, Sungjin Park, Seong Min Kim, Hye Eun Lee, Minjae Joo, Kyoung Kon Kim, Doojin Kim, Dong Hae Chung, Jae Been Im, Jaehun Jung, Seung Kak Shin, Byung-Chul Oh, Cheolsoo Choi, Seungyoon Nam, Dae Ho Lee

**Affiliations:** 1 Department of Health Sciences and Technology, Gachon Advanced Institute for Health Sciences and Technology (GAIHST), Gachon University, Incheon 21999, Korea; ronnyron@gachon.ac.kr (A.P.); chan7844@gachon.ac.kr (M.J.); dlawoqls1230@naver.com (J.B.I.); 2 Department of Radiology, Gil Medical Center, Gachon University College of Medicine, Incheon 21565, Korea; sjchoi@gilhospital.com; 3 Department of Genome Medicine and Science, AI Convergence Center for Genome Medicine, Gachon Institute of Genome Medicine and Science, Gachon University Gil Medical Center, Gachon University College of Medicine, Incheon 21565, Korea; oscarpark@gachon.ac.kr; 4 Department of Surgery, Gil Medical Center, Gachon University College of Medicine, Incheon 21565, Korea; seongmin_kim@gilhospital.com (S.M.K.); drkdj@gilhospital.com (D.K.); 5 Department of Internal Medicine, Gil Medical Center, Gachon University College of Medicine, Incheon 21565, Korea; esthel0513@gmail.com (H.E.L.); puressk@gilhospital.com (S.K.S.); cschoi@gilhospital.com (C.C.); 6 Department of Family Medicine, Gil Medical Center, Gachon University College of Medicine, Incheon 21565, Korea; zaduplum@gilhospital.com; 7 Department of Pathology, Gil Medical Center, Gachon University College of Medicine, Incheon 21565, Korea; dhchung@gilhospital.com; 8 Department of Preventive Medicine, Gachon University College of Medicine, Incheon 21565, Korea; eastside1st@gachon.ac.kr; 9 Department of Physiology, Lee Gil Ya Cancer and Diabetes Institute, Gachon University College of Medicine, Incheon 21999, Korea; bcoh@gachon.ac.kr

**Keywords:** nonalcoholic fatty liver disease, aldo-keto reductase family 1 member B10, biomarkers, diagnosis

## Abstract

We found several blood biomarkers through computational secretome analyses, including aldo-keto reductase family 1 member B10 (AKR1B10), which reflected the progression of nonalcoholic fatty liver disease (NAFLD). After confirming that hepatic AKR1B10 reflected the progression of NAFLD in a subgroup with NAFLD, we evaluated the diagnostic accuracy of plasma AKR1B10 and other biomarkers for the diagnosis of nonalcoholic steatohepatitis (NASH) and fibrosis in replication cohort. We enrolled healthy control subjects and patients with biopsy-proven NAFLD (*n* = 102) and evaluated the performance of various diagnostic markers. Plasma AKR1B10 performed well in the diagnosis of NASH with an area under the receiver operating characteristic (AUROC) curve of 0.834 and a cutoff value of 1078.2 pg/mL, as well as advanced fibrosis (AUROC curve value of 0.914 and cutoff level 1078.2 pg/mL), with further improvement in combination with C3. When we monitored a subgroup of obese patients who underwent bariatric surgery (*n* = 35), plasma AKR1B10 decreased dramatically, and 40.0% of patients with NASH at baseline showed a decrease in plasma AKR1B10 levels to below the cutoff level after the surgery. In an independent validation study, we proved that plasma AKR1B10 was a specific biomarker of NAFLD progression across varying degrees of renal dysfunction. Despite perfect correlation between plasma and serum levels of AKR1B10 in paired sample analysis, its serum level was 1.4-fold higher than that in plasma. Plasma AKR1B10 alone and in combination with C3 could be a useful noninvasive biomarker for the diagnosis of NASH and hepatic fibrosis.

## 1. Introduction

Nonalcoholic fatty liver disease (NAFLD) is becoming a more prevalent and burdensome metabolic disease [1]. The prevalence of NAFLD in obese subjects undergoing bariatric surgery can be more than 95%, with 20–98% having steatohepatitis (NASH) and 10–94.1% having fibrosis [2,3,4]. Bariatric surgery can lead to the resolution of NASH in up to 60–84% of patients [2,5]. However, in the majority of cases, serial liver biopsies are impractical for longitudinal monitoring of NAFLD.

Several blood markers for NASH have been introduced but are not universally accepted: aspartate aminotransferase (AST)/alanine aminotransferase (ALT) ratio, cytokeratin 18, and other predictive models using clinical and laboratory values [6,7,8]. Several magnetic resonance (MR)-based parameters, including the proton density fat fraction measured by MR imaging (MRI-PDFF), two-dimensional (2D) or 3D liver stiffness measurement (LSM) by MR elastography (MRE), and iron-corrected T1 relaxation time, have been introduced for the prediction of NASH and are awaiting further validation [8,9,10,11].

To identify blood biomarkers for NASH and related hepatic fibrosis, we profiled secretome genes from public gene expression datasets that included NAFLD and its progressive hepatic manifestations, such as advanced fibrosis, cirrhosis, and hepatocellular carcinoma (HCC). We found that aldo-keto reductase family 1 member B10 (AKR1B10) could be biomarker(s) of NAFLD progression. AKR1B10 catalyzes the reduction of retinaldehyde to retinol and the detoxification of harmful metabolites, including reactive aldehydes and ketones [12,13]. Although AKR1B10 is profusely expressed in epithelial tissues of the digestive tract, with a low level in the liver [12], it has been reported that this enzyme is upregulated in some cancers, including HCC [14,15]. In addition, recent studies have reported that this enzyme is also upregulated in NASH with stage 4 fibrosis (F4) [16,17,18,19]. However, the diagnostic performance of plasma AKR1B10 for NASH and its changes after therapeutic intervention for NAFLD need further systematic validation.

Considering the secretory characteristics of AKR1B10 and the spectrum of NAFLD ranging from NAFL to NASH to cirrhosis and HCC, we aimed to evaluate the feasibility of AKR1B10 as a blood biomarker of NASH and fibrosis with parallel analyses of other blood and imaging biomarkers. We also evaluated whether the AKR1B10-based approach is applicable to the monitoring of NAFLD in obese patients undergoing laparoscopic sleeve gastrectomy (LSG). Additionally, we performed a validation cohort study to determine whether this blood biomarker had NAFLD-specific performance independent of renal dysfunction.

## 2. Results

### 2.1. Identification of DEGs for Secretory Proteins in NAFLD and HCC

**Figure 1:**
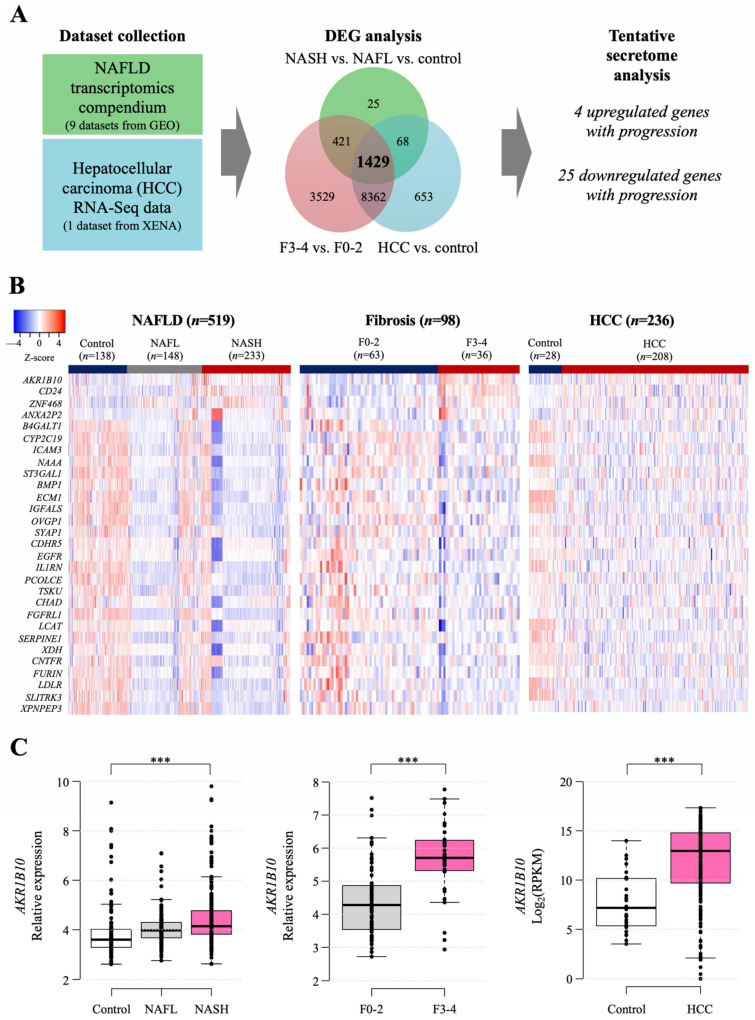
Computational identification of the tentative secretome reflecting NAFLD progression that was available from the analyses of public gene datasets. (**A**) Workflow of secretome identification by analyzing public datasets of NAFLD- and HCC-related genes. Detailed information on the datasets is described in Section 4 and presented in Supplementary Table S1. (**B**) Heatmap of putative secretory biomarker genes. Stepwise upregulated (upper 4 listed genes) and downregulated common genes (lower 25 listed genes) according to disease progression. (**C**) Hepatic *AKR1B10* expression according to the progression of NAFLD was divided into 3 categories of disease progression. ***, *p* < 0.001.

The workflow for searching tentative secretory proteins from public datasets of NAFLD- and HCC-related human hepatic gene expression is presented in Figure 1A, and detailed information on the datasets is summarized in Supplementary Table S1. The NAFLD data were obtained from nine Gene Expression Omnibus (GEO) datasets, including 138 normal samples, 148 NAFL samples, 233 NASH samples, and 98 with NAFLD with hepatic fibrosis samples (Supplementary Table S1), while the data of 236 HCC samples were downloaded from UCSC-XENA (Figure 1A). We categorized the NAFLD-related data into three datasets to identify differentially expressed genes (DEGs) in each dataset: control, NAFL, and NASH; fibrosis F0-2 and F3-4 grades; and control (nontumor tissue) and HCC. Then, we identified 1429 common DEGs. We selected genes that encode secretory proteins and that showed stepwise increasing or decreasing expression patterns according to disease progression (henceforth, disease progression biomarker candidates [DPBCs]). As a result, 4 upregulated secretory DPBCs (*AKR1B10*, *ANXA2P2*, *CD24*, and *ZNF468*) and 25 downregulated secretory DPBCs (as listed in Figure 1B) were finally obtained.

Among the four increased targets, as shown in the following results, AKR1B10 gene and protein showed significant and consistent changes with respect to NAFLD progression. Thus, we decided to focus more on AKR1B10 in this study. The increasing patterns of *AKR1B10* in each comparison are presented graphically in Figure 1C, and those of the other genes are presented in Supplementary Figure S1.

### 2.2. The Relationship between NAFLD Progression and AKR1B10 Expression Levels

**Figure 2:**
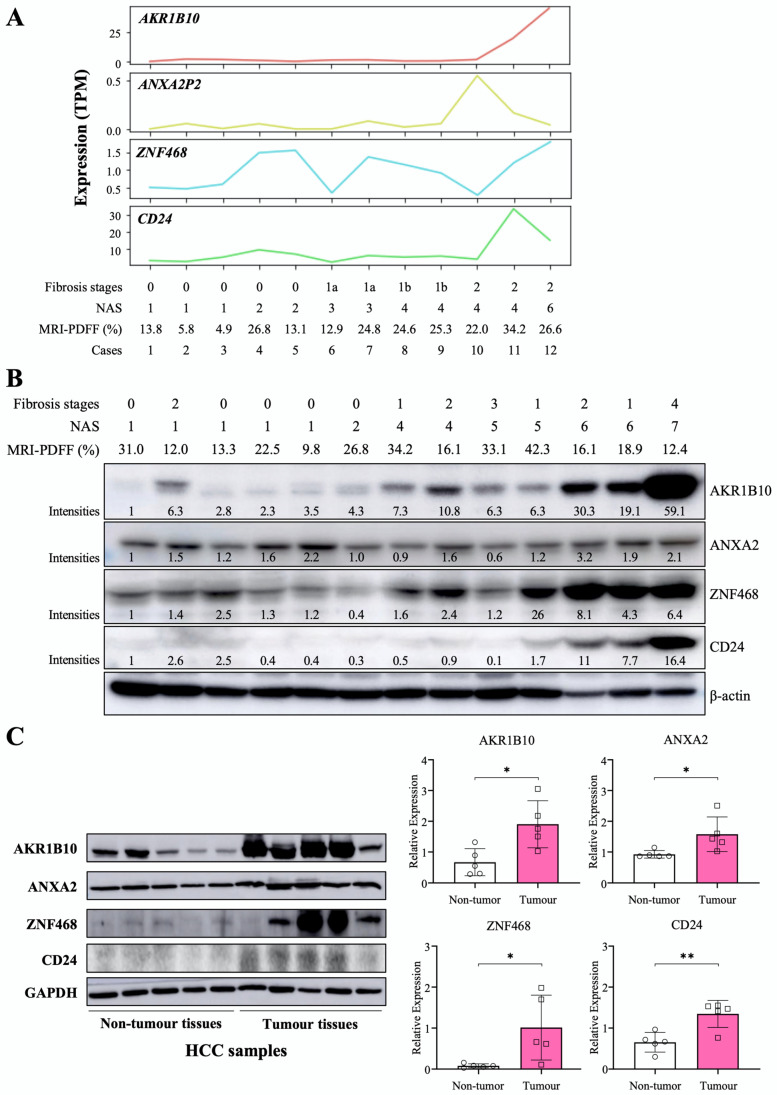
Hepatic expression of the AKR1B10 gene and protein in patients with NAFLD and HCC. (**A**) RNA sequencing data showing 4 upregulated common DEGs in study subjects with a spectrum of NAFLD progression based on the NAFLD activity score (NAS) and fibrosis stage (*n* = 12). TPM, transcripts per million. (**B**) Immunoblotting analysis and semiquantification of AKR1B10, ZNF468, annexin A2 (ANXA2), and CD24 protein expression in liver tissues from independently selected study subjects with a spectrum of NAFLD progression (*n* = 13). Note that protein expression levels normalized by β-actin expression were expressed relative to the corrected intensity values of the first lanes in each band. (**C**) Immunoblotting analysis and semiquantification of AKR1B10, ZNF468, ANXA2, and CD24 protein expression in nontumor and tumor tissues of liver samples from patients with HCC (*n* = 5). Band intensities of blots were normalized with respect to the signal intensities of the loading internal control (β-actin or GAPDH) detected on the same blots. *—*p* < 0.05; **—*p* < 0.01. Note: because commercial antibody to annexin A2P2 (ANXA2P2) was not available, immunoblotting on ANXA2 protein, a paralogous protein of ANXA2P2, was performed.

We performed RNA-Seq for liver tissues from selected participants with a spectrum of NAFLD progression based on NAFLD activity score (NAS) and fibrosis stages (*n* = 12). In another independent small group, we evaluated AKR1B10 protein expression in liver tissues with a NAS ranging from 1 to 7 (*n* = 13). AKR1B10 mRNA and protein showed a consistently increasing trend as the NAS increased (Figure 2). Other DPBCs showed variable patterns (Figure 2 and Supplementary Figure S2). In addition, when we evaluated liver tissues of five patients with HCC, the protein expression of AKR1B10, ZNF468, annexin A2 (ANXA2), and CD24 was increased in tumor tissue compared with surrounding nontumor tissues (Figure 2C).

### 2.3. AKR1B10 Expression in Publicly Available scRNA-Seq Datasets of Human Liver

We evaluated the expression level of *AKR1B10* in individual cells by analyzing two human scRNA-Seq datasets, namely, GSE129933 [20], and GSE136103 [21]. The two scRNA-Seq datasets were for lymphatic endothelial cell-enriched nonparenchymal liver cells from healthy control subjects and patients with NASH [20] and for CD45-negative cells that are found in healthy and NAFLD-related cirrhotic livers [21] (Supplementary Table S2; Supplementary Figure S3A). *AKR1B10* was upregulated in hepatocytes from patients with NASH (GSE129933) or cirrhosis (GSE136103) compared with hepatocytes from their respective control subjects (Supplementary Figure S3B,C). In addition, its expression was also increased in hepatic stellate cells in patients with cirrhosis compared with control subjects (GSE136103) (Supplementary Figure S3C).

### 2.4. The Discriminative Performance of Plasma AKR1B10 for the Identification of NASH and Fibrosis

In the replication study, we analyzed 102 subjects as patients with NAFL (*n* = 28) and with NASH (*n* = 50) on liver biopsy and healthy control subjects (*n* = 24) (Table 1), who were selected from a pooled cohort of 158 subjects (Supplementary Table S3) to evaluate the diagnostic accuracy of plasma AKR1B10 and other blood biomarkers and imaging biomarkers, such as MR-measured parameters (PDFF and LSM) and transient elastography (TE) parameters [controlled attenuation parameter (CAP) and LSM].

**Figure 3:**
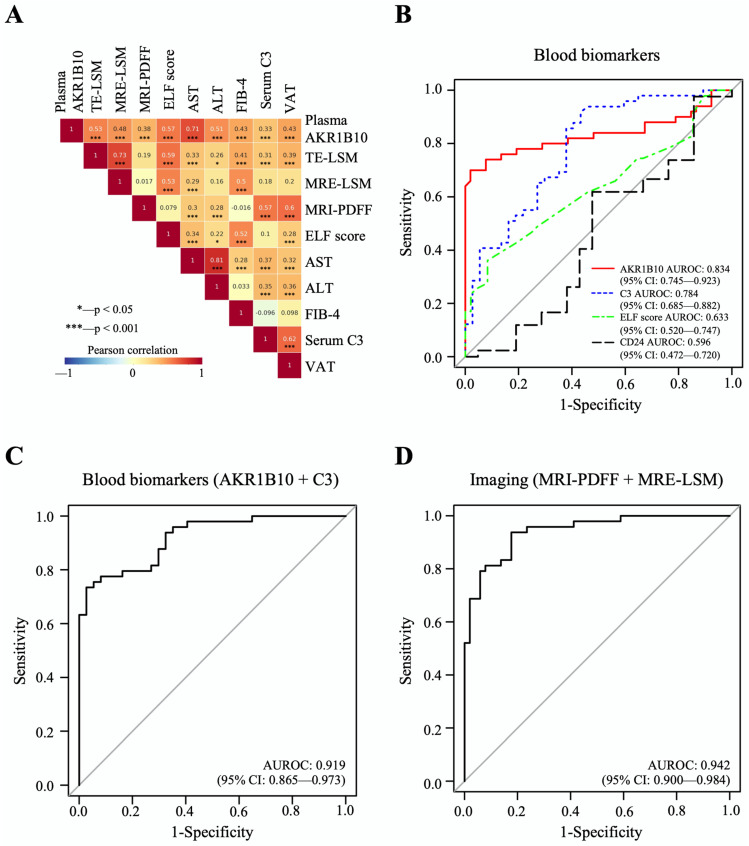
Correlations between major parameters and AUROC curves of biomarkers for the detection of NASH. (**A**) The results of Pearson’s correlation analyses between major parameters. (**B**–**D**), The predictive performance of AKR1B10 and other biomarkers for NASH vs. normal/NAFL.

The mean plasma level of AKR1B10 was higher in patients with NASH (7629.7 ± 7045.1) than in patients with NAFL (421.7 ± 235.8 pg/mL, *p* < 0.01) and healthy control subjects (549.8 ± 235.2 pg/mL, *p* < 0.01). Plasma AKR1B10 showed relatively strong correlations with AST, TE- and MRE-LSM, ALT, enhanced liver fibrosis (ELF) score, FIB-4, and visceral adipose tissue (VAT) and significant correlations with other parameters (Figure 3A and Supplementary Table S4). To see whether plasma AKR1B10 is still a good biomarker of NASH and fibrosis, independent of obesity, insulin resistance, and other parameters, we performed multiple logistic regression analysis against the outcomes, having age, sex, BMI, HOMA-IR, ALT, MRI-PDFF, and AKR1B10 (log-transformed) as covariates. The results showed that plasma AKR1B10 is an independent predictor of NASH (Supplementary Table S5) and advanced fibrosis (Supplementary Table S6).

The area under the receiver operating characteristic curve (AUROC) of plasma AKR1B10 for the identification of patients with NASH was 0.834, with an optimal cutoff value of 1078.2 pg/mL (Table 2 and Figure 3B–D). The results of AUROC were 0.850, with an optimal cutoff value of 1078.2 pg/mL, when we analyzed only subjects with biopsy results (*n* = 79) (Supplementary Table S7). For advanced fibrosis, plasma AKR1B10 showed excellent performance at a much higher cutoff level (AUROC 0.914 and cutoff level 1078.2 pg/mL) (Table 2). We also calculated AUROCs for other clinically available markers: C3 as a blood biomarker; combined MR-based parameters (MRI-PDFF and MRE-LSM); and combined TE-based parameters (CAP and TE-LSM) (Table 2). Plasma AKR1B10 alone did not outperform MRI-PDFF/MRE-LSM. However, its combination with serum C3 further improved the AUROC curve value (0.919) (Table 2 and Figure 3B–D).

When we set the plasma AKR1B10 cutoff level at 1078.2 pg/mL considering the AUROC of AKR1B10-based model, 4.1% (1/24) of the healthy control subjects, no patients with NAFL (0/28), and specifically 70% of patients with NASH (35/50) had a plasma AKR1B10 value above the threshold.

### 2.5. Changes in Plasma AKR1B10 and Other NAFLD-Related Parameters before and after Bariatric Surgery

We also analyzed a subgroup of bariatric surgery applicants; among 53 participants who underwent bariatric surgery (LSG), 35 patients completed prescheduled follow-up examinations after the surgery while the other subjects were lost during observation or had not yet completed follow-up examinations yet. The characteristics of this subgroup of bariatric surgery subjects before and after LSG are provided in Table 3. At enrollment, the mean age of the LSG cohort was 34.3 ± 7.6 years with a prevalence of females (85.7%). The preoperative mean body mass index (BMI) was 38.1 ± 5.0 kg/m2, with a mean body weight of 102.0 ± 15.4 kg. The mean baseline plasma AKR1B10 was 3243.8 ± 5381.0 pg/mL, while hepatic PDFF and MRE-LSM were 19.1 ± 9.3% and 3.1 ± 0.9 kPa, respectively.

**Figure 4:**
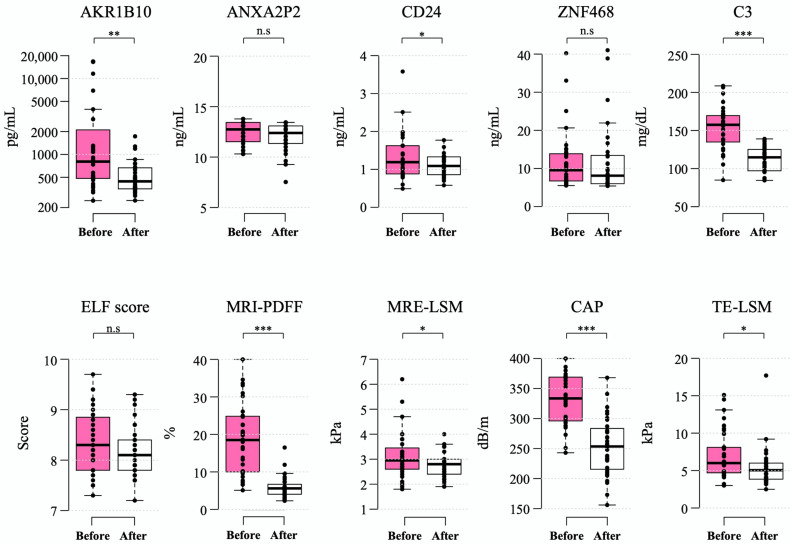
Changes in biomarkers 6–12 months after bariatric surgery. Box-whisker plots are shown, with the bottom and top of the box representing the 25th and 75th percentiles, respectively, and the middle line representing the median. The whiskers extend to the 5th and 95th percentiles, and outliers are presented as dots. *—*p* < 0.05; **—*p* < 0.01; ***—*p* < 0.001; and n.s.—not significant.

After a median of 6.5 (interquartile range 6.2–6.8) months after bariatric surgery, the mean levels of plasma AKR1B10 decreased to 529.0 ± 350.5 pg/mL (mean decrease, 2714.8 pg/mL). These changes were accompanied by a marked decrease in BMI (mean decrease, 9.4 kg/m2), body weight (mean decrease, 24.8 kg), and MRI-PDFF (mean decrease 13.1% point) as well as a significant improvement in other parameters. However, the ELF score did not decrease significantly after bariatric surgery (Table 3 and Figure 4).

All of the patients undergoing the surgery had steatosis at baseline and, after LSG in 40.0% of patients, their hepatic PDFF was normalized to a value less than 5%. The fraction of patients with NAFLD and a high plasma AKR1B10 level (≥1078.2 pg/mL) was 40.0% (14 of 35 patients) in this subgroup, in which 71.4% of them (10/14) showed a decrease to less than the AKR1B10 cutoff level after LSG. A total of 20 out of 35 patients had NAS ≥ 3 on liver biopsy during LSG. Among these 20 patients with NASH on liver biopsy, 8 patients (40.0%) had plasma AKR1B10 that decreased to below the cutoff level after LSG, while 7 patients (35.0%) had both MRI-PDFF and MRE-LSM values that decreased to below their respective cutoffs after bariatric surgery.

### 2.6. Plasma and Serum AKR1B10 Measurement in the Validation Cohort

**Figure 5:**
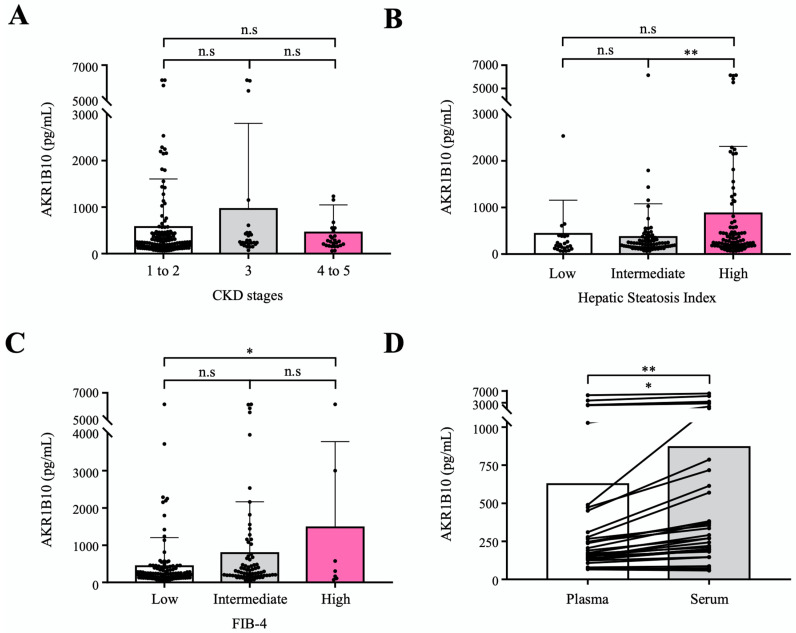
Validation of plasma AKR1B10 as a NAFLD progression marker in an independent cohort with a broad range of eGFRs. (**A**) Plasma AKR1B10 levels according to CKD status (*n* = 195). (**B**) and (**C**) Plasma AKR1B10 levels according to the likelihood of hepatic steatosis and advanced fibrosis based on HSI and FIB-4 score systems (*n* = 195). (**D**) Comparison of paired plasma and serum measurements of AKR1B10 in selected patients across a range of eGFRs. *—*p* < 0.05; **—*p* < 0.01; and n.s.—not significant.

To evaluate the circulating AKR1B10 as a biomarker of NAFLD progression relatively independent of renal dysfunction, we measured plasma AKR1B10 levels in an independent cohort that included healthy control subjects and patients with type 2 diabetes mellitus (T2DM) and a spectrum of chronic kidney disease (CKD). A total of 195 subjects were included in the validation cohort study in which 30 healthy control subjects (aged 57.0 ± 6.5) and 165 patients with T2DM (aged 60.6 ± 7.6) were subjected to routine clinical biochemistry and the measurement of plasma AKR1B10 levels. The characteristics of the study subjects are presented in Supplementary Table S8. The mean estimated glomerular filtration rate (eGFR) of study subjects was 77.0 ± 30.3 mL/min per 1.73 m^2^, ranging from 6.3 to 119.7 mL/min per 1.73 m^2^. Because the feasibility of circulating biomarkers can be affected by renal dysfunction, with AKR1B10 being expressed in renal tissue [22], we intended to assess the performance of plasma AKR1B10 across CKD stages in the prediction of hepatic steatosis and advanced fibrosis, which were estimated and categorized by using the eGFR, hepatic steatosis index (HSI), and FIB-4, respectively. The mean plasma AKR1B10 levels were 283.1 ± 329.1 pg/mL in healthy control subjects and 667.5 ± 1215.2 pg/mL in patients with T2DM (*p* = 0.087). Plasma levels of AKR1B10 were not different among the 3 CKD category subgroups (CKD stages 1–2, CKD stage 3, and CKD stages 4–5; Figure 5A), whereas plasma AKR1B10 levels showed increasing patterns with higher levels of HSI and FIB-4 categories (Figure 5B,C). In addition, plasma AKR1B10 did not correlate with eGFR (r = −0.063, *p* = 0.380). Significantly high levels of plasma AKR1B10 were also observed in higher grade HSI and FIB-4 categories in our replication study (Supplementary Figure S4).

Considering the clinical application in patients with NAFLD, we measured AKR1B10 levels in paired plasma and serum samples simultaneously in 32 selected subjects after stratification according to eGFR. Plasma and serum levels of AKR1B10 showed a perfect correlation (r = 0.988, *p* < 0.001). However, the serum level of AKR1B10 was 1.39-fold higher than that of plasma (868.7 ± 1477.0 pg/mL vs. 625.7 ± 1191.5 pg/mL, *p* < 0.01) (Figure 5D).

## 3. Discussion

In the present study, by analyzing publicly available datasets of NAFLD-related genes, we searched tentative secretory proteins that reflect the progression of NAFLD with a consistent and stepwise increasing pattern for the diagnosis of NASH. We found four secretory proteins (AKR1B10, annexin A2P2, ZNF468, and CD24) and we showed through our own cohort study that plasma AKR1B10 could be a clinically applicable biomarker for NASH (AUROC 0.834) and for advanced fibrosis (AUROC 0.914) at a cutoff level (≥1078.2 pg/mL). We applied our protocol to monitor NAFLD in patients undergoing bariatric surgery, showing that plasma AKR1B10 levels decreased markedly after surgery. In the independent validation study, we also proved that plasma AKR1B10 was a specific biomarker of NAFLD progression that was not influenced by renal function. Furthermore, we also showed that by measuring AKR1B10 in paired plasma and serum samples of selected patients with a broad range of eGFRs, there was a perfect correlation between plasma and serum levels of AKR1B10; however, there were approximately 1.4-fold higher levels in serum than in plasma.

AKR1B10 has been reported to be increased in some cancers, including HCC [12,14,15,19,23] as well as chronic liver diseases including NASH [17,18,19,24,25] and fibrosis/cirrhosis [16]. AKR1B10 is a retinaldehyde reductase and is also involved in the regulation of fatty acid synthesis by stabilizing acetyl-CoA carboxylase-α [12,26]. Interestingly, hepatic stellate cells store 50–80% of body retinol in the form of retinyl esters [12]. Thus, AKR1B10 seems to have important pathophysiological implications in NAFLD and its progression and complications.

Independent of previous studies on AKR1B10 and liver diseases [14,15,16,17,18,27], we found AKR1B10 via computational secretome analysis based on the DEGs according to NAFLD progression, and then showed that its gene and protein expression in the liver increased consistently and gradually according to disease progression via multiple approaches in our replication and validation cohorts.

It has yet to be clarified how and at what stage AKR1B10 is involved in the progression of NAFLD. There may be several pathways by which this subtype of enzyme of the AKR subfamily affects NAFLD progression: (i) reduced protection against oxidative stress due to increased aldose reductase activity and increased advanced glycation end-products [28]; (ii) low intracellular level of retinoic acid due to AKR1B10-mediated reduction of retinaldehyde to retinol [12,29]; and (iii) reduced production of natural peroxisome proliferator-activated receptor-γ ligands via the diversion of prostaglandin (PG) D2 toward PG F2α and away from PG J2 [30]. In line with our results, a recent study showed that *AKR1B10* and *SPP1* were closely related to progression and prognosis in normal-NAFL-NASH-HCC progression [18]. However, we did not observe a consistent and stepwise increase in *SPP1* expression in liver samples from our study subjects (data not shown). There have also been some studies that showed an increase in serum AKR1B10 in fibrosing NASH [16,17]. However, there is no study showing a marked decrease in plasma AKR1B10 values after bariatric surgery in patients with NAFLD. Normal ranges of plasma/serum AKR1B10 have been different between studies, including the present study and cutoff levels of AKR1B10 for the prediction of NASH and/or advanced fibrosis and HCC varied and overlapped between different studies [14,16]. Kanno et al. reported that serum AKR1B10 at a cutoff level of 1003 pg/mL was predictive of stage 4 fibrosis in NASH [16]. Thus, although AKR1B10 may be a molecular marker reflecting the progression of NAFLD ranging from steatohepatitis to HCC, further clinical studies are required to standardize the measurement of plasma/serum AKR1B10. In the present study, we showed that the diagnostic threshold values of plasma AKR1B10 for NASH and advanced fibrosis were similar and showed that the serum AKR1B10 level was consistently higher than the plasma level of AKR1B10, which needs to be considered in the clinical application of this biomarker. Furthermore, serial measurements of circulating AKR1B10 after various therapeutic interventions, including lifestyle modification, in patients with NAFLD can be more informative.

Plasma AKR1B10 can be used in combination with other blood biomarkers, depending on the clinical context. Complement activation and C3 deposition in the liver were shown to be associated with excessive fat accumulation, hepatocyte apoptosis, and hepatic neutrophil sequestration and increased with NAFLD severity [31,32]. In this study, when AKR1B10 was combined with C3, the AUROC value for NASH differential diagnosis was significantly improved.

Various MR techniques and parameters can be applied in diagnosing and monitoring the spectrum of NAFLD [33,34]. Additionally, a multiparametric MR-based approach enabled us to assess NAFLD as well as other body factors (VAT, subcutaneous adipose tissue [SAT], and pancreatic fat). MRI-PDFF is expressed as a percentage and an accurate and precise method for the measurement of fat across the entire biological range of hepatic steatosis [35,36]. A significant proportion of patients with NASH have hepatic fibrosis and the fibrosis stage correlates significantly with NASH activity [21,37,38,39]. Thus, several studies have shown the performance of MRE in the discrimination between isolated steatosis and NASH with an AUROC ranging from 0.70 to 0.93 [37,40,41,42]. A recent study showed that a protocol combining 3D-MRE measuring LSM at 60 Hz, the damping ratio at 40 Hz, and MRI-PDFF could detect NASH with an AUROC of 0.73 [10]. In the present study, reflecting those previous reports, the MRI-PDFF and MRE-LSM combination performed well in predicting NASH and advanced fibrosis (Table 2). However, cost and availability of MR are major limitations.

Many studies have shown that bariatric surgery, including LSG, causes a significant improvement in NASH and fibrosis [5,43]. A recent elegant study involving 71 patients with Child-A NASH-related cirrhosis showed that fibrosis regressed in 67.7% of the patients after LSG, while NASH improved in 60.6% of the patients [5]. In the present study, a BMI reduction of 9.4 kg/m^2^ could be achieved at 6.5 months after LSG. It was reported that a mean BMI reduction of 12 kg/m^2^ or more after bariatric surgery was more likely to be associated with the resolution of NASH without progression of fibrosis [2]. In the present study, we did not perform follow-up liver biopsy after bariatric surgery. Among 20 patients with baseline NAS ≥ 3 at the time of LSG, 40.0% had decreased plasma AKR1B10 levels to below 1078.2 pg/mL and 35.0% had decreased MR-parameters (PDFF and LSM) to below their cutoff levels after LSG, indicating that NASH can be improved significantly in approximately 35–67% of patients who received LSG depending on the assessment protocol.

Our study has several limitations. First, our study is a single-centre study in Korea and AUROCs may be affected by the disease spectrum of the study cohort. However, we systematically proved the feasibility of AKR1B10 as a biomarker of NAFLD progression through diverse experimental approaches and validation cohort study. Second, the validation study was not based on liver biopsy results. However, HSI and FIB-4 are useful scoring systems for steatosis and advanced hepatic fibrosis [44,45]. In the validation cohort study, we showed that plasma AKR1B10 levels were a specific NAFLD progression marker across a wide range of eGFRs. Third, follow-up liver biopsy was not performed in patients with biopsy-proven NAFLD because of practical reasons.

In conclusion, plasma AKR1B10 alone and in combination with C3, as a noninvasive biomarker, performed well in the identification of NASH and advanced fibrosis, and is helpful for the longitudinal monitoring of the progression of or improvement in NAFLD. Further validation studies and the standardization of plasma AKR1B10 measurement would be important for its clinical application.

## 4. Materials and Methods

### 4.1. Data Acquisition and the Selection of Genes Encoding Secretory Proteins

We obtained and processed publicly available gene expression datasets of liver tissues for NAFLD [46] and HCC [47] (Figure 1A and Supplementary Table S1). Please see the Supplementary Method S1 for more details on the datasets and their processing. We identified common differentially expressed genes (DEGs) in the following three comparisons for disease progression: (i) NAFLD progression (control, NAFL and NASH); (ii) hepatic fibrosis progression (F0-2 and F3-4); and (iii) control and HCC. For each comparison, statistical significance for a DEG was set to a Benjamini-Hochberg false discovery rate of less than 0.1. Subsequently, common DEGs among all three comparisons were obtained. Then, we further narrowed the common DEGs down by visual inspection, such that each DEG had a gradually increasing or decreasing pattern of expression according to disease progression in all three comparisons. These DPBC genes, were considered for the subsequent computational secretome analysis, in which The Human Protein Atlas, MetazSecKB, and VerSeDa databases were used to identify genes, encoding secretory proteins, from the DPBCs [48,49,50,51].

### 4.2. Single-Cell RNA-Seq (scRNA-Seq) Data Analysis Using Publicly Available Datasets

To inspect the expression levels of the upregulated DPBCs at the single-cell level in liver tissue samples from control subjects and patients with NAFLD, we analyzed two scRNA-Seq datasets (GSE129933 [20] and GSE136103 [21]) from GEO, which included data on nonparenchymal cells and CD45-negative cells from liver tissue samples, respectively (Supplementary Table S2), as detailed in the Supplementary Method S2.

### 4.3. Study Subjects and Design

We evaluated the diagnostic accuracy of plasma AKR1B10 and other blood biomarkers and imaging biomarkers, such as MR-measured parameters (PDFF and LSM) and TE parameters (CAP and LSM), to detect NASH (NAS ≥ 3) on histology with a subscore of 1 or higher for each subcomponent (steatosis, hepatocyte ballooning, and lobular inflammation) and advanced fibrosis (F3-4) [52] in this replication cohort study, which included healthy control subjects with or without liver biopsy results and patients with NAFLD with liver biopsy results within 3 months prior to enrollment. Those subjects were eligible from a pooled cohort of 4 parent studies: (1) a study involving healthy control subjects; (2) a study involving patients with NAFLD who volunteered for an MR-based NAFLD study, with some having liver biopsy data; (3) a study involving a bariatric surgery cohort with liver biopsy results; and (4) a study of living liver transplant donors with liver biopsy results. A flow chart of the cohort study is presented in Supplementary Figure S5.

The age of the study subjects was required to be between 19 and 70 years of age. The healthy controls were required to have no evidence of NAFLD on liver biopsy or an MRI-PDFF less than 5% and normal results of liver function and other biochemical tests even without liver biopsy: in males, AST < 40 U/L, and ALT < 35 U/L; in females, AST < 40 U/L, and ALT < 25 U/L. Exclusion criteria were excessive alcohol consumption (alcohol intake >20 g/day for women and >30 g/day for men), evidence of another coexistent liver or biliary disease except for NAFLD, use of medications known to cause secondary hepatic steatosis within 1 year, contraindications for MR studies, or any conditions that might affect patient competence or participation as determined by the opinion of the principal investigator. We analyzed a subgroup of bariatric surgery applicants who completed baseline and follow-up examinations 6–12 months after metabolic surgery between March 2018 and April 2021. The study protocols were in accordance with the Declaration of Helsinki and were approved by the institutional review board at the Gil Medical Center. All participants provided written informed consent and all parent studies were registered at https://cris.nih.go.kr (last accessed on 22 March 2022) in accordance with the International Clinical Trials Registry Platform.

In addition, we retrospectively evaluated the expression of some secretory proteins in tumor and nontumor liver tissues from patients with HCC (*n* = 5; 4 NAFLD-related cases and one HCV-related case). The biospecimen and data used in this study were provided by Gachon University Gil Medical Center Bio Bank (No. GBB2020-02). The study protocols were in accordance with the Declaration of Helsinki and were approved by the institutional review board at the Gil Medical Center. All participants provided written informed consent to deposit and use their tissues in the biobank for research purposes.

### 4.4. Clinical and Laboratory Evaluation

An array of clinical and laboratory data were collected in the replication study, as detailed previously [31]. After an overnight fast, blood samples were collected on the same day or within days of the imaging studies or several days before liver biopsy for various markers and routine biochemical tests, which included liver function tests, glucose, insulin, a complete blood count with a platelet count, albumin, haemoglobin A1c (HbA1c), lipid panel, AKR1B10, zinc finger protein (ZNF) 468, CD24, complement factors C3 and C4, and the ELF test [31]. Sample processing and measurement details are available in the Supplementary Method S3. Body fat and lean body mass were measured using the dual energy X-ray absorptiometry (DXA) technique (GE Healthcare, Wauwatosa, WI, USA) on the same day as the imaging studies. Other clinical indices and scores were calculated as previously described [31].

### 4.5. Imaging Biomarker Studies

The hepatic and pancreatic MRI-PDFF, hepatic R2* relaxation rate of the water protons representing liver iron content, the VAT and SAT areas, and MRE-LSM were measured with a 3-T scanner (MAGNETOM Skyra; Siemens Healthineers, Erlangen, Germany) using an 18-channel body matrix coil and table-mounted 32-channel spine matrix coil [31], as detailed in the Supplementary Method S4. TE was performed using FibroScan 502 (Echosens, Paris, France) by a trained technician blinded to the clinical and histological data, as previously described [31].

### 4.6. Liver Tissue Sampling and Analyses

Liver biopsy and tissue sampling were performed during bariatric surgery, donor liver resection for living liver transplantation, and percutaneous liver biopsy procedures due to abnormal liver function. The liver tissues were analyzed as described in more detail in the Supplementary Method S5. Histological scoring, including NAS and fibrosis staging, was performed using the Nonalcoholic Steatohepatitis Clinical Research Network histologic scoring system [52]. RNA sequencing analyses were performed for 12 liver samples from study subjects with a spectrum of NAFLD. Transcripts per million mapped reads were used for mRNA expression. The RNA-Seq data were deposited to the NCBI BioProject (accession: PRJNA716432) available at https://www.ncbi.nlm.nih.gov/sra/PRJNA716432 (accessed on 22 March 2022). Additionally, liver tissues from study participants with a spectrum of NAFLD (*n* = 13) and those from the biobank of the hospital were processed for immunoblotting analyses of AKR1B10 and other proteins as described in more detail in the Supplementary Method S5.

### 4.7. Validation of Plasma AKR1B10 as a Biomarker in an Independent Cohort

We measured plasma AKR1B10 levels in an independent cohort that included healthy control subjects and patients with T2DM and a spectrum of CKD. Detailed information about this cohort study is described in the Supplementary Method S6. We intended (1) to validate plasma AKR1B10 as a NAFLD progression biomarker that is clinically useful; (2) to compare AKR1B10 levels in paired plasma and serum samples in stratified subgroups; and (3) to prove that plasma AKR1B10 is elevated independent of renal impairment in patients with progressive NAFLD. In this validation cohort, we divided study participants into subgroups in each of three separate analyses, according to CKD stages by the eGFR, the risk of steatosis by the HIS, and the risk of liver fibrosis by the FIB-4 index. Based on previously known low and high cutoff values for the prediction of steatosis and advanced fibrosis [31], we divided participants into 3 groups each: low, intermediate, and high likelihood for steatosis (HSI values <30, 30–36, and >36, respectively) [44] and for advanced fibrosis (FIB-4 indices <1.3, 1.3–2.67, and >2.67, respectively) [45]. We followed the same ethical rules, and the study was registered as stated for the replication study in Section 4.3.

### 4.8. Statistics

Categorical variables were compared as counts and percentages and associations were tested using the chi-squared or Fisher’s exact test. Continuous variables were reported as the mean ± standard deviation (SD) or medians with 25th–75th percentiles when appropriate, and differences between groups were analyzed using Student’s *t*-test (two-tailed) or the Mann–Whitney U test, paired *t*-test, or one-way ANOVA followed by Tukey–Kramer’s multiple comparisons post hoc test as appropriate. Univariate and multiple logistic regression analyses to assess for the potential predicting factors of NASH and advanced liver fibrosis were performed. Correlations were evaluated using Pearson’s correlation coefficients. The performance of diagnostic markers was assessed by AUROC, sensitivity, specificity, positive predictive value (PPV), and negative predictive value (NPV). For each AUROC, 95% confidence intervals (CIs) were measured using its standard error. The AUROC and the optimal thresholds were obtained by the multipleROC package in R. Paired-sample *t*-tests or Wilcoxon rank-sum tests were used to compare the data before and after surgery and the differences between plasma and serum samples. All reported *p* values are two-sided and considered statistically significant at <0.05. Statistical analyses were performed using R software/environment (R version 2.9.1).

## Figures and Tables

**Table 1:** Demographic and clinical characteristics of the study subjects.

Characteristics	Control (*n* = 24)	NAFL (*n* = 28)	NASH/ Cirrhosis (*n* = 50)	*p* Values
Age (years)	36 (15.7)	35.1 (7.9)	35.3 (12.0)	0.96
Sex (male/female)	17/7	5/23 ^††^	12/38 ^##^	<0.001
Weight (kg)	66.7 (11.4)	90.9 (18.3) ^††^	103.1 (20.7) ^##,^ *	<0.001
BMI (kg/m^2^)	23.0 (3.1)	33.5 (6.2) ^††^	38.1 (6.5) ^##,^ *	<0.001
WC (cm)	80.2 (8.0)	104.0 (14.4) ^††^	112.3 (13.5) ^##,^ *	<0.001
SBP (mmHg)	130.2 (16.9)	121.4 (13.7) ^†^	127.3 (14.8)	0.09
DBP (mmHg)	83.4 (11.9)	84.2 (10.8)	86.3 (10.2)	0.50
AST (U/L)	20.8 (5.6)	28.5 (29.2)	65.4 (49.7) ^##,^ **	<0.001
ALT (U/L)	18.4 (7.1)	44.5 (84.5)	83.7 (59.8) ^##,^ *	<0.001
GGT (U/L)	18.6 (8.2)	36.2 (33.1) ^†^	84.4 (118.7) ^##,^ *	<0.05
Total cholesterol (mg/dL)	188.2 (36.5)	194.8 (41.5)	210.3 (35.2) ^#^	<0.05
HDL cholesterol (mg/dL)	59.3 (15.1)	51.7 (12.9)	49.4 (17.3) ^#^	<0.05
Triglycerides (mg/dL)	100.1 (45.9)	152.6 (138.1)	173.9 (66.0) ^##^	<0.05
WBC (× 10^9^/L)	5.2 (1.7)	7.2 (2.2) ^†^	8.2 (1.9) ^##,^ *	<0.001
Platelets (× 10^9^/L)	229.0 (52.4)	303.8 (66.0) ^††^	318.7 (101.5) ^##^	<0.001
HbA1c (%)	5.4 (0.4)	5.9 (1.8)	6.6 (1.7) ^##^	<0.05
Glucose (mg/dL)	89.3 (6.9)	109.8 (50.7) ^†^	122.4 (53.8) ^##^	<0.05
Insulin (μU/mL)	6.7 (3.9)	24.0 (40.5) ^†^	26.6 (24.3) ^##^	<0.05
HOMA-IR	1.5 (1.0)	6.8 (14.1)	8.9 (8.7) ^##^	<0.05
C3 (mg/dL)	104.7 (16.3)	142.3 (32.5) ^††^	166.2 (33.0) ^##,^ *	<0.001
C4 (mg/dL)	26.5 (5.6)	34.9 (11.9) ^†^	35.9 (11.7) ^##^	<0.05
ANXA2P2 (ng/mL)	57.8 (22.6)	23.5 (20.6) ^††^	28.1 (24.7) ^##^	<0.001
CD24 (ng/mL)	2.6 (5.4)	2.0 (4.1)	1.3 (1.1)	0.32
ZNF468 (ng/mL)	19.5 (13.8)	13.0 (8.4)	11.5 (8.3) ^#^	<0.05
AKR1B10 (pg/mL)	549.8 (235.2)	421.7 (235.8)	7629.7 (7045.1) ^##,^ **	<0.001
Hepatic steatosis index	12.7 (14.7)	75.6 (26.7) ^††^	91.9 (11.2) ^##,^ *	<0.001
FIB-4	0.8 (0.4)	0.6 (0.2) ^†^	1.2 (2.2) *	0.18
ELF score	8.2 (0.8)	8.3 (0.6)	8.8 (1.1) ^#,^ *	<0.05
CAP (dB/m)	216.5 (37.9)	304.5 (52.1) ^††^	342.3 (47.6) ^##,^ *	<0.001
TE-LSM (kPa)	3.8 (0.9)	6.4 (3.7) ^†^	11.6 (10.3) ^##,^ *	<0.001
Liver MRI-PDFF (%)	3.4 (0.8)	11.3 (7.1) ^††^	21.8 (9.3) ^##,^ **	<0.001
MRE-LSM (kPa)	3.2 (0.6)	2.8 (0.6) ^†^	3.8 (1.5) ^#,^ **	<0.05
Liver R2* (s^−1^)	43.9 (6.4)	51.0 (10.1) ^†^	61.4 (13.0) ^##,^ **	<0.001
DXA total body fat (%)	24.9 (9.8)	46.4 (7.4) ^††^	49.1 (6.8) ^##^	<0.001
DXA total muscle (kg)	46.7 (13.8)	45.9 (7.3)	47.2 (15.4)	0.91
MRI-VAT area (cm^2^)	62.7 (35.4)	144.6 (57.8) ^††^	189.2 (72.0) ^##,^ *	<0.001
MRI-SAT area (cm^2^)	121.0 (51.4)	320.4 (103.7) ^††^	388.1 (126.1) ^##,^ *	<0.001

Data are expressed as the mean (SD) or n (%), unless otherwise specified. Abbreviations: WC—waist circumference; SBP—systolic blood pressure; DBP—diastolic blood pressure; GGT—γ-glutamyl transpeptidase; HDL—high-density lipoprotein; HOMA-IR—homoeostatic model assessment of insulin resistance; DXA—dual-energy X-ray absorptiometry; R2*—apparent transverse relaxation rate; SAT—subcutaneous adipose tissue; VAT—visceral adipose tissue; ^†^—*p* < 0.05; ^††^—*p* < 0.01; vs. healthy controls; ^#^—*p* < 0.05; ^##^—*p* < 0.01; vs. healthy controls; *—*p* < 0.05; **—*p* < 0.01; vs. NAFL.

**Table 2:** The performance of plasma AKR1B10 and other blood and imaging biomarkers and their cutoff values for the diagnosis of NASH and advanced fibrosis (F3-4) (*n* = 102) *.

NASH						
Parameters/Applications	AUROC (95% CI)	Cutoff	Sensitivity (%)	Specificity (%)	PPV (%)	NPV(%)
AKR1B10 (pg/mL)	0.834 (0.745–0.923)	1078.2	70.0	98.1	97.2	77.3
C3 (mg/dL)	0.784 (0.685–0.882)	124.1	91.8	56.8	73.8	84.0
ELF score	0.633 (0.520–0.747)	9.0	36.2	91.7	81.0	53.1
MRI-PDFF (%) + MRE-LSM (kPa)	0.942 (0.900–0.984)	11.5/3.3	93.8	82.4	83.3	93.3
CAP (dB/m) + TE-LSM (kPa)	0.871 (0.799–0.942)	268/5.3	100.0	66.7	74.6	100.0
AKR1B10 (pg/mL) + C3 (mg/dL)	0.919 (0.865–0.973)	641.5/174.9	73.5	97.3	97.3	73.5
**Advanced hepatic fibrosis (F3-4)**						
AKR1B10 (pg/mL)	0.914 (0.847–0.981)	1078.2	100.0	71.7	27.8	100
ELF score	0.833 (0.686–0.979)	8.9	77.8	74.4	24.1	97.0
MRE-LSM (kPa)	0.981 (0.955–1.000)	4.0	100.0	90.0	50.0	100.0
TE-LSM (kPa)	0.877 (0.769–0.985)	8.1	90.0	77.6	32.1	98.5

* In a total of 102 subjects in the pooled cohort, 50 patients had NAS ≥ 3, while 10 patients had advanced hepatic fibrosis (F ≥ 3). Abbreviations: CI—confidence interval; LR—likelihood ratio; NPV—negative predictive value; PPV—positive predictive value.

**Table 3:** Follow-up interval changes in the characteristics of the subgroup of patients who underwent bariatric surgery (*n* = 35) *.

Characteristics	Before Surgery	After Surgery	Mean Difference	*p* Values
Age (years)	34.3 (7.6)	34.8 (7.7)	−0.5	<0.001
Sex (male/female)	5/30	5/30	NA	NA
Weight (kg)	102.0 (15.4)	77.3 (14.5)	24.8	<0.001
BMI (kg/m^2^)	38.1 (5.0)	28.7 (4.7)	9.4	<0.001
Waist circumference (cm)	111.9 (9.8)	90.5 (9.4)	21.4	<0.001
SBP (mmHg)	126.8 (14.3)	114.2 (13.0)	12.6	<0.001
DBP (mmHg)	88.1 (10.2)	81.8 (9.2)	6.3	<0.05
AST (U/L)	39.0 (26.2)	19.2 (11.7)	19.8	<0.001
ALT (U/L)	56.1 (39.3)	16.9 (9.3)	39.2	<0.001
GGT (U/L)	58.0 (44.0)	21.8 (14.5)	36.2	<0.001
Total cholesterol (mg/dL)	205.4 (37.3)	192.1 (27.1)	13.3	<0.05
HDL-C (mg/dL)	46.8 (7.4)	52.1 (12.6)	−5.3	<0.05
Triglycerides (mg/dL)	163.5 (78.0)	103.3 (40.5)	60.1	<0.001
White blood cell (×10^9^/L)	8.1 (2.1)	6.6 (2.1)	1.5	<0.001
Platelets (×10^9^/L)	338.8 (96.9)	292.3 (74.2)	46.6	<0.001
Hemoglobin A1c (%)	6.1 (1.3)	5.5 (1.3)	0.7	<0.001
Glucose (mg/dL)	110.8 (32.7)	96.4 (37.2)	14.4	<0.05
Insulin (μU/mL)	24.5 (15.4)	9.1 (4.1)	15.4	<0.001
HOMA-IR	7.1 (6.0)	2.0 (1.0)	5.1	<0.001
C4 (mg/dL)	37.1 (11.2)	31.7 (9.4)	5.4	<0.001
Hepatic steatosis index	51.6 (5.8)	38.1 (4.6)	13.5	<0.001
FIB-4	0.56 (0.29)	0.61 (0.33)	−0.05	0.41
DXA total body fat (%)	49.9 (5.5)	42.1 (8.8)	7.8	<0.001
DXA total muscle (kg)	48.9 (7.9)	42.4 (7.7)	6.6	<0.001
Liver R2* (s^−1^)	59.2 (11.2)	45.8 (9.8)	13.4	<0.001
MRI-VAT area (cm^2^)	175.2 (68.2)	93.9 (31.3)	81.3	<0.001
MRI-SAT area fat (cm^2^)	383.8 (111.0)	250.2 (80.4)	133.6	<0.001
Pancreas MRI-PDFF (%)	6.9 (5.3)	3.7 (4.0)	3.2	<0.001

* Among 53 patients who underwent bariatric surgery, 35 patients finished prescheduled follow-up at a median of 6.5 months after the surgery. Data are expressed as the mean (SD) or *n* (%), unless otherwise specified. Abbreviations: WC—waist circumference; SBP—systolic blood pressure; DBP—diastolic blood pressure; GGT—γ-glutamyl transpeptidase; HDL—high-density lipoprotein; HOMA-IR—homoeostatic model assessment of insulin resistance; DXA—dual-energy X-ray absorptiometry; R2*—apparent transverse relaxation rate; SAT—subcutaneous adipose tissue; VAT—visceral adipose tissue. *—*p* < 0.05 vs. before surgery.

## Data Availability

All data generated in this study are included in this manuscript. The RNA-Seq data for 12 liver samples in this study were deposited to the NCBI BioProject (accession: PRJNA716432) available at https://www.ncbi.nlm.nih.gov/sra/PRJNA716432 (accessed on 22 March 2022).

## Supplementary Materials

The following supporting information can be downloaded at: https://www.mdpi.com/article/10.3390/ijms23095035/s1.

## Abbreviations

AKR1B10—aldo-keto reductase family 1 member B10; ALT—alanine aminotransferase; AST—aspartate aminotransferase; AUROC—area under the receiver operating characteristic; BMI—body mass index; CAP—controlled attenuation parameter; C3—complement component 3; CKD—chronic kidney disease; DEGs—differentially expressed genes; DPBCs—disease progression biomarker candidates; ELF—enhanced liver fibrosis; HSI—hepatic steatosis index; LSG—laparoscopic sleeve gastrectomy; LSM—liver stiffness measurement; MRE—magnetic resonance elastography; MRI—magnetic resonance imaging; PDFF—proton density fat fraction; NAFLD—nonalcoholic fatty liver disease; NASH—nonalcoholic steatohepatitis; SD—standard deviation; TE—transient elastography.
