# Supplementary material for: Plasma Aldo-Keto Reductase Family 1 Member B10 as a Biomarker Performs Well in the Diagnosis of Nonalcoholic Steatohepatitis and Fibrosis
Source: Int J Mol Sci. 2022 May 1;23(9):5035. doi: 10.3390/ijms23095035 (PMC9101253; doi:10.3390/ijms23095035)
Supplement: Supplementary file 1 [file ijms-23-05035-s001.zip › ijms-1672504-supplementary.pdf]

Plasma aldo-keto reductase family 1 member B10 as a diagnostic biomarker of nonalcoholic steatohepatitis and fibrosis

Aron Park, Seung Joon Choi, and Sungjin Park et al.

Supplementary Information

Contents

Supplementary Method S1. Data acquisition and the selection of genes encoding secretory proteins

Supplementary Method S2. Single-cell RNA-Seq (scRNA-Seq) data analysis using publicly available datasets

Supplementary Method S3. Clinical and laboratory evaluation

Supplementary Method S4. Magnetic resonance (MR) studies

Supplementary Method S5. Liver tissue sampling and analyses

Supplementary Method S6. Validation of plasma AKR1B10 as a biomarker in an independent cohort

Supplementary Table S1. Information on public datasets used in the present study for NAFLD gene expression compendium.

Supplementary Table S2. Dataset description for publicly available single-cell RNA sequencing of human liver tissues.

Supplementary Table S3. Demographic and clinical characteristics of subjects included in the pooled cohort.

Supplementary Table S4. Pearson's correlation coefficients between plasma AKR1B10 and other parameters.

Supplementary Table S5. Univariate and multiple regression analyses in the prediction of NASH (*n*=102).

Supplementary Table S6. Univariate and multiple regression analyses in the prediction of advanced fibrosis (F3-4) (*n*=102).

Supplementary Table S7. The performance of plasma AKR1B10 and other blood and imaging biomarkers and their cutoff values for the diagnosis of NASH in subjects with liver biopsy results (*n*=79).

Supplementary Table S8. Demographic and clinical characteristics of the study subjects in the validation cohort.

Supplementary Figure S1. Other putative secretory biomarker genes that showed a common stepwise upregulation according to NAFLD progression.

Supplementary Figure S2. RNA sequencing (RNA-Seq) data of 25 downregulated common differentially expressed genes listed in Figure 1B.

Supplementary Figure S3. The expression of AKR1B10 at the single cell level: Analyses of public single-cell RNA sequencing (scRNA-Seq) datasets.

Supplementary Figure S4. Relationship between plasma AKR1B10 and NAFLD-related clinical scores in the replication study (n=102).

Supplementary Figure S5. Flowchart showing enrollment of study subjects in the replication cohort study.

Supplementary Methods

Method S1. Data acquisition and the selection of genes encoding secretory proteins

We collected hepatic gene expression microarray datasets of NAFLD with the search terms "NAFLD", "nonalcoholic fatty liver disease", and "NASH" in the Gene Expression Omnibus (GEO) as of September 30, 2019. Among the datasets, we selected datasets with the Affymetrix platform. As a result, 9 datasets were obtained as summarized in Figure 1A and Supplementary Table S1. Robust multiarray analysis (RMA) normalization was performed on each dataset using the oligo package in R. All of the datasets were combined, and batch effect removal was conducted by ComBat function in the Surrogate Variable Analysis (SVA) package in R. As a result, the combined gene expression compendium for NAFLD was obtained. In addition, we downloaded the HCC RNA sequencing (RNA-Seq) dataset of The Cancer Genome Atlas (n=423, released on October 13, 2017) obtained from the UCSC XENA database. Excluding patients with viral hepatitis, 236 samples (28 controls and 208 HCC cases) were analyzed in this study (Figure 1A).

Method S2. Single-cell RNA-Seq (scRNA-Seq) data analysis using publicly available datasets

In GSE129933, the samples were hepatic nonparenchymal cells from human liver tissues, while in GSE136103, CD45(-) cells from human liver tissue samples were analyzed (Supplementary Table S2). The Python scanpy package was used for scRNA-Seq analysis. For preprocessing of the GSE129933 dataset, we removed cells with less than 300 or greater than 2,500 uniquely expressed genes, which followed the same criteria as in the publication of the dataset, with default parameter values of the package. Only genes expressed in greater than or equal to 3 cells were used for further analysis. Cells were also discarded if

mitochondrial gene percentages were greater than 5%. The unique molecular barcode counts were natural log transformed and normalized for scaling the sequencing depth with 10,000 molecules per cell. In the GSE129933 dataset, the cell type annotation of cell clustering followed the same annotation as it was in the publication of the dataset. For preprocessing of the GSE136103 dataset, we removed cells with less than 300 uniquely expressed genes following the same criteria as in the publication of the dataset. Only genes expressed in greater or equal to 3 cells were used for further analysis. Cells were also discarded if mitochondrial gene percentages were greater than 30%, which followed the same criteria as it was in the publication of the dataset. The unique molecular barcode counts were natural log transformed and normalized for scaling the sequencing depth with 10,000 molecules per cell. We first identified highly variable positive (upregulated) genes detected with default parameters of the scanpy package. We performed PCA using the highly variable genes and significant principal components (PCs). With 10 PCs, clusters were identified within a resolution of 0.5. In GSE136103, Leiden clustering was performed [1], a total of 16 clusters were obtained, and significantly expressed marker genes in each cell type cluster were selected by the Wilcoxon rank-sum test [2]. In GSE136103, the cell type was annotated by referring to the marker genes in The Human Protein Atlas [3].

Method S3. Clinical and laboratory evaluation

All blood samples were originally processed into serum and plasma and stored frozen at -80°C. Currently available commercial kits were used for the measurement of plasma levels of AKR1B10 (Abcam, Cambridge, UK) and zinc finger protein (ZNF) 468, CD24 and putative annexin A2-like protein (ANXA2P2) (Mybiosource, San Diego, CA), serum complement factors C3 and C4 (turbidimetric immunoassay Tina-quant C3c and C4, Roche Diagnostics

Ltd., Rotkreuz, Switzerland), and the enhanced liver fibrosis (ELF) test (The ADVIA Centaur Enhanced Liver Fibrosis Test, Siemens Healthcare, Erlangen, Germany).

Method S4. Magnetic resonance (MR) studies

The MR studies were performed with a 3-T scanner (MAGNETOM Skyra; Siemens Healthineers, Erlangen, Germany) using an 18-channel body matrix coil and table-mounted 32-channel spine matrix coil. To quantify the fat content and R2* relaxation rate of the water protons in the liver, we used a multiecho 3D gradient-echo sequence to obtain MRI-PDFF from a single breath-hold acquisition. In addition, we measured the pancreatic fat content in the regions of interest at the head, neck, and tail of the pancreas as well as visceral adipose tissue (VAT) and subcutaneous adipose tissue (SAT) areas at the L3-L4 disc level [4].

The 2D spin-echo echo-planar imaging MRE sequence (Work-In-Progress package, Siemens Healthineers, Erlangen, Germany) was also performed in the same session to obtain LSM values for the liver parenchyma, as previously described.

Method S5. Liver tissue sampling and analyses

Bariatric surgery with liver biopsy on segment III or IV of the liver was performed by a surgeon (S. M. K.), while liver biopsy sampling from living liver transplantation donors was performed during liver resection by another surgeon (D. J. K.). Some liver biopsy results included in our replication study were from subjects who underwent percutaneous liver biopsy due to abnormal liver function. A pathologist (D. H. C.) who was blinded to the patients' clinical and radiologic results assessed the stained specimens. Histological scoring including the NAS was performed using the Nonalcoholic Steatohepatitis Clinical Research Network histologic scoring system. Fibrosis was staged from F0 to F4. A part of each liver

tissue was also snap frozen in liquid nitrogen and stored at -80°C until additional analysis. For RNA sequencing analysis, paired-end RNA libraries for 12 liver samples from study subjects with a spectrum of NAFLD were constructed and sequenced at Macrogen, Inc. (Seoul, Korea), using the TruSeq Stranded mRNA LT Sample Prep Kit (Illumina, San Diego, CA, USA), according to the manufacturer's protocol. The raw read data were trimmed by Trimmomatic v0.38 [5]; quality control was conducted by FastQC v0.11.7; the trimmed read data were aligned with the human reference genome (GRCh38) by HISAT2 v2.1.0 [6]; and the aligned read data were assembled by StringTie v2.1.3b [7]. Transcripts per million mapped reads were used for mRNA expression. The RNA-Seq data for 12 liver samples in this study were deposited to the NCBI BioProject (accession: PRJNA716432) available at https://www.ncbi.nlm.nih.gov/sra/PRJNA716432.

For immunoblotting analysis, human liver tissues were homogenized in lysis buffer (CST, Danvers, MA, USA) and centrifuged at 15,000 rpm for 10 min at 4 °C; supernatants were collected for protein isolation and prepared for sodium dodecyl sulfate (SDS)-polyacrylamide gel electrophoresis (PAGE). Equal volumes of sample were resolved by SDS-PAGE and electrotransferred to polyvinylidene difluoride membranes (GE Healthcare, Chicago, IL, USA). The membranes were blocked to prevent nonspecific binding and incubated with the corresponding primary antibody and secondary antibody conjugated to horseradish peroxidase. Bands were visualized using an enhanced chemiluminescence system. The primary and secondary antibodies used in these experiments were as follows: anti-AKR1B10 (ab96417), anti-annexin-2 (ANXA2) (ab41803), anti-CD24 (ab179821), and goat anti-rabbit IgG (ab6721) antibodies from Abcam (Cambridge, UK); anti-beta-actin (8457s) and GAPDH (D16H11) antibodies from CST (Danvers, MA, USA); and anti-ZNF468 antibody (PA5-69738) from Invitrogen (Carlsbad, CA, USA).

Method S6. Validation of plasma AKR1B10 as a biomarker in an independent cohort

Study population and protocol: Patients with T2DM (*n*=165) and healthy control subjects (*n*=30) were involved in this cross-sectional study. Male or female patients aged 30-75 years with T2DM diagnosed at the age of 30 years or later and healthy controls without T2DM of similar ages were eligible to participate in the study. Patients with T2DM in this cohort had a spectrum of renal function from normoalbuminuria and normal kidney function to rapidly progressive diabetic kidney disease to CKD stage 4 or 5. Patients were required to have haemoglobin A1c value of 11% or less, a urine albumin-to-creatinine ratio (UACR) of less than 3500 mg/g, a haemoglobin level of 11 g/dL or more, and a serum albumin level of 3.6 g/dL or more at screening. If patients were on antihypertensive medication or had been taking an ACE inhibitor or angiotensin II receptor blocker (ARB) for diabetic kidney disease, they were only included if they had been on a stable dose for at least 2 weeks. Patients with poor BP control or those taking 4 or more antihypertensive drugs were excluded. Exclusion criteria were renal replacement therapy, CKD presumed to be a result of nondiabetic causes, subjects with a single kidney, and known liver cirrhosis or viral hepatitis. Patients with a history of acute kidney injury, a long-term history of the use of renal toxic drugs for more than 3 months, or end-stage renal disease were also excluded from the study.

Study participants were instructed to visit the laboratory after fasting overnight. The serum and plasma samples from each subject were aliquoted and stored at -80 °C until further use. Some of study results in this cohort on the relationship between serum dipeptidyl peptidase 4 activity and plasma catecholamine levels were published previously [8].

Measurements and calculations: Plasma AKR1B10 levels were measured in all participants, while serum AKR1B10 levels were measured in selected subjects who were stratified based on renal function (*n*=32). The estimated glomerular filtration rate (eGFR) was calculated by using the CKD Epidemiology Collaboration equation. To estimate the

likelihood of hepatic steatosis and advanced fibrosis in the study subjects, clinical scoring systems, the hepatic steatosis index (HSI) and FIB-4 were calculated.

Supplementary Table S1. Information on public datasets used in the present study for NAFLD gene expression compendium.

GEO accession	The number of study subjects in the dataset			References
	Total	Controls	Cases	
**Datasets for NAFLD (Control, NAFL, and NASH)**				
**GSE37031**	Controls: 7<br>Cases: 8	Healthy: 7	NASH: 8	López-Vicario et al. [9]
**GSE48452**	Controls: 28<br>Cases: 26	Healthy:12<br>Obese:16	NAFL: 9<br>NASH: 17	Ahrens et al. [10]
**GSE58979**	Cases: 26		NASH: 26	du Plessis et al. [11]
**GSE61260**	Controls: 62<br>Cases: 24	Healthy: 38<br>Obese: 24	NASH: 24	Horvath et al. [12]
**GSE63067**	Controls: 7<br>Cases: 11	Healthy: 7	NAFL: 2<br>NASH: 9	Frades et al. [13]
**GSE66676**	Controls: 34<br>Cases: 33	Obese: 34	NAFL: 26<br>NASH: 7	Xanthakos et al. [14]
**GSE83452**	Cases: 115		NAFL: 98<br>NASH: 126	Lefebvre et al. [15]
**GSE106737**	Cases: 29		NAFL: 13<br>NASH: 16	Haas et al. [16]
**Datasets for NAFLD with hepatic fibrosis (F0-2 and F3-4)**				
**GSE48452**	Controls: 22<br>Cases: 4	F0-2: 22	F3-4: 4	Ahrens et al. [10]
**GSE31803**	Controls: 40<br>Cases: 32	F0-2: 40	F3-4: 32	Murphy et al. [17]

Supplementary Table S2. Dataset description for publicly available single-cell RNA sequencing of human liver tissues.

GEO accession	Number of samples	Controls	Cases	References
**GSE129933**	Controls: 2<br>Case: 1	Nondiseased, LEC-enriched hepatic nonparenchymal cells	NASH, LEC-enriched hepatic nonparenchymal cells	Tamburini et al. [18]
**GSE136103**	Controls: 6<br>Cases: 2	Healthy, CD45(-) cells	Cirrhotic (NAFLD), CD45(-) cells	Ramachandran et al. [19]

Abbreviation: LEC, lymphatic endothelial cells.

Supplementary Table S3. Demographic and clinical characteristics of subjects included in the pooled cohort.

Characteristics	Healthy volunteers (*n*=24)	Patients with NAFLD (*n*=134)	Pooled cohort (*n*=158)
**Age (years)**	36.0 (15.7)	38.4 (13.5)	38.1 (13.8)
**Sex (male/female)**	17/7	48/84	65/91
**Weight (kg)**	66.7 (11.4)	94.8 (22.7)	90.5 (23.6)
**BMI (kg/m^2)**	23.0 (3.1)	34.3 (7.2)	32.5 (7.8)
**WC (cm)**	80.2 (8.0)	106.7 (15.5)	102.7 (17.4)
**SBP (mmHg)**	130.2 (16.9)	126.4 (14.9)	127.0 (15.2)
**DBP (mmHg)**	83.4 (11.9)	85.3 (10.1)	85.0 (10.4)
**AST (U/L)**	20.8 (5.6)	48.0 (43.5)	43.9 (41.3)
**ALT (U/L)**	18.4 (7.1)	67.5 (70.4)	60.1 (67.2)
**GGT (U/L)**	18.6 (8.2)	62.8 (82.3)	56.0 (77.5)
**Total cholesterol (mg/dL)**	188.2 (36.5)	196.1 (42.3)	194.9 (41.5)
**HDL cholesterol (mg/dL)**	59.3 (15.1)	48.8 (14.1)	50.4 (14.7)
**Triglycerides (mg/dL)**	100.1 (45.9)	159.1 (95.5)	150.1 (92.1)
**WBC (x10^9/L)**	5.2 (1.7)	7.4 (2.1)	7.1 (2.2)
**Platelets (x10^9/L)**	229.0 (52.4)	287.8 (87.2)	278.9 (85.4)
**HbA1c (%)**	5.4 (0.4)	6.5 (1.7)	6.3 (1.6)
**Glucose (mg/dL)**	89.3 (6.9)	115.4 (44.9)	111.4 (42.5)
**Insulin (μU/mL)**	6.7 (3.9)	23.0 (25.5)	20.5 (24.2)
**HOMA-IR**	1.5 (1.0)	7.0 (9.0)	6.1 (8.5)
**ANXA2P2 (ng/mL)**	57.8 (22.6)	38.0 (30.8)	41.5 (30.4)
**CD24 (ng/mL)**	2.6 (5.4)	1.4 (2.1)	1.6 (2.9)
**ZNF468 (ng/mL)**	19.5 (13.8)	13.6 (10.2)	14.7 (11.1)
**AKR1B10 (pg/mL)**	549.8 (235.2)	4872.1 (6395.4)	4131.1 (6043.3)
**Hepatic steatosis index**	30.9 (4.3)	47.1 (9.4)	44.6 (10.6)
**FIB-4**	0.82 (0.45)	1.03 (1.44)	1.00 (1.34)
**ELF score**	8.2 (0.8)	8.8 (1.0)	8.7 (1.0)
**CAP (dB/m)**	216.5 (37.9)	320.9 (56.8)	303.8 (66.5)
**TE-LSM (kPa)**	3.8 (0.9)	9.1 (9.5)	8.2 (8.9)
**DXA total body fat (%)**	24.9 (9.8)	44.2 (9.6)	41.2 (11.8)
**DXA total muscle (kg)**	46.7 (13.8)	44.8 (17.5)	45.1 (17.0)
**Liver MRI-PDFF (%)**	3.4 (0.8)	16.7 (9.5)	14.7 (10.0)
**MRE-LSM (kPa)**	3.2 (0.6)	3.6 (1.3)	3.5 (1.2)
**Liver R2* (s^{-1})**	43.9 (6.4)	56.8 (13.2)	54.8 (13.2)
**MRI-VAT area (cm^2)**	62.7 (35.4)	177.5 (83.6)	159.7 (88.5)
**MRI-SAT area (cm^2)**	121.0 (51.4)	313.1 (131.4)	283.3 (140.9)

Data are expressed as the mean (standard deviation) or n (%), unless otherwise specified. Abbreviations: SBP, systolic blood pressure; DBP, diastolic blood pressure; GGT, γ-glutamyl transpeptidase; HDL, high-density lipoprotein; HOMA-IR, homeostatic model assessment of insulin resistance; NFS, NAFLD fibrosis score; DXA, dual-energy X-ray absorptiometry; R2*, apparent transverse relaxation rate; SAT; subcutaneous adipose tissue; VAT; visceral adipose tissue.

Supplementary Table S4. Pearson's correlation coefficients between plasma AKR1B10 and other parameters*.

Parameters	r	p	Parameters	r	p
**Age (years)**	0.147	< 0.001	**Insulin (μU/mL)**	0.204	0.042
**WC (cm)**	0.372	< 0.001	**HOMA-IR**	0.221	0.027
**BMI (kg/m^2)**	0.362	< 0.001	**Total Cholesterol (mg/dL)**	0.108	0.283
**WBC (x10^9/L)**	0.218	0.028	**Triglycerides (mg/dL)**	0.136	0.176
**Glucose (mg/dL)**	0.051	0.614	**DXA total body fat (%)**	0.231	0.020
**HbA1c (%)**	0.130	0.197	**CAP (dB/m)**	0.345	0.001
**GGT (U/L)**	0.330	0.001	**MRI-SAT area (cm^2)**	0.276	0.006
**LDH (U/L)**	0.417	< 0.001	**Creatinine (mg/dL)**	-0.175	0.115

*Please see also Figure 3A. **Abbreviations:** WC, waist circumference; WBC, white blood cell; HbA1c, haemoglobin A1c; GGT, γ-glutamyl transpeptidase; LDH, lactic dehydrogenase; HOMA-IR, homeostatic model assessment of insulin resistance; DXA, dual-energy X-ray absorptiometry; SAT; subcutaneous adipose tissue.

Supplementary Table S5. Univariate and multiple regression analyses in the prediction of NASH (*n* = 102).

Parameters	Univariate analysis		Multiple logistic regression analysis	
	ORs (95% CIs)	*P* values	ORs (95% CIs)	*P* values
Age (years)	0.998 (0.966 to 1.031)	0.92		
Sex	1.719 (0.695 to 4.252)	0.23		
BMI (kg/m^2)	1.203 (1.119 to 1.129)	< 0.001	1.128 (1.008 to 1.263)	< 0.05
HOMA-IR	1.086 (1.001 to 1.179)	< 0.05	0.977 (0.923 to 1.034)	0.42
ALT (U/L)	1.023 (1.010 to 1.036)	< 0.001	1.001 (0.990 to 1.011)	0.83
MRI-PDFF (%)	1.238 (1.143 to 1.340)	< 0.001	1.178 (1.062 to 1.306)	< 0.05
*AKR1B10 (pg/mL)	16.994 (5.225 to 55.275)	< 0.001	12.787 (2.918 to 56.037)	< 0.001

Abbreviations: OR, odds ratio; CI, confidence interval. *Test on $\log_{10}$-transformed values.

Supplementary Table S6. Univariate and multiple regression analyses in the prediction of advanced fibrosis (F3-4) (*n* = 102).

Parameters	Univariate analysis		Multiple logistic regression analysis	
	ORs (95% CIs)	*P* values	ORs (95% CIs)	*P* values
Age (years)	1.086 (1.029 to 1.458)	< 0.05	1.097 (1.023 to 1.175)	< 0.05
Sex	0.725 (0.190 to 2.766)	0.64		
BMI (kg/m^2)	1.066 (0.981 to 1.159)	0.11		
HOMA-IR	0.998 (0.932 to 1.068)	0.95		
ALT (U/L)	1.008 (1.000 to 1.016)	< 0.05	1.008 (0.997 to 1.019)	0.13
MRI-PDFF (%)	1.032 (0.974 to 1.093)	0.28		
*AKR1B10 (pg/mL)	14.814 (2.980 to 73.642)	< 0.001	8.829 (1.868 to 41.730)	< 0.05

Abbreviations: OR, odds ratio; CI, confidence interval. *Test on log_{10}-transformed values.

Supplementary Table S7. The performance of plasma AKR1B10 and other blood and imaging biomarkers and their cutoff values for the diagnosis of NASH in subjects with liver biopsy results (*n* = 79).

**Parameters**	**AUROC (95% CI)**	**Cutoff**	**Sensitivity (%)**	**Specificity (%)**	**PPV (%)**	**NPV(%)**
AKR1B10 (pg/mL)	0.850 (0.766-0.935)	1078.2	70.0	100.0	100.0	65.9
C3 (mg/dL)	0.703 (0.579-0.827)	174.9	40.8	92.3	90.9	45.3
ALT (U/L)	0.809 (0.709-0.909)	32	78.0	75.9	84.8	66.7
ELF score	0.647 (0.521-0.773)	8.9	42.6	88.5	87.0	46.0
MRI-PDFF (%) + MRE-LSM (kPa)	0.919 (0.860-0.978)	30.2/1.9	72.9	100.0	100.0	68.3
CAP (dB/m) + TE-LSM (kPa)	0.760 (0.641-0.879)	367/4.4	63.8	80.0	85.7	57.1
AKR1B10 + C3	0.909 (0.846-0.972)	1303.84/115.9	77.6	92.3	95.0	68.6

In a total of 79 subjects in the pooled cohort, 50 patients had NAS ≥ 3. Abbreviations: CI, confidence interval; LR, likelihood ratio; NPV, negative predictive value; PPV, positive predictive value.

Supplementary Table S8. Demographic and clinical characteristics of the study subjects in the validation cohort.

Characteristics	Healthy volunteers (*n*=30)	Patients with T2DM (*n*=165)	Pooled cohort (*n*=195)
**Age (years)**	57.0 (6.5)	60.6 (7.6)	60.0 (7.5)
**Sex (male/female)**	18/12	99/66	117/78
**Creatinine (mg/dL)**	0.8 (0.2)	1.3 (0.9)	1.2 (0.9)
**eGFR (mL/min per 1.73 m^2)**	98.0 (8.7)	73.2 (31.2)	77.0 (30.3)
**Hepatic steatosis index**	32.5 (4.3)	36.4 (4.6)	35.8 (4.7)
**FIB-4**	1.47 (0.77)	1.36 (1.03)	1.38 (0.99)
**Height (cm)**	166.8 (9.1)	164.3 (8.3)	164.6 (8.4)
**Weight (kg)**	67.2 (11.6)	68.6 (11.2)	68.4 (11.3)
**BMI (kg/m^2)**	24.1 (3.4)	25.4 (3.4)	25.2 (3.4)
**SBP (mmHg)**	131.5 (18.0)	137.2 (18.3)	136.3 (18.3)
**DBP (mmHg)**	83.3 (9.6)	81.3 (11.4)	81.6 (11.2)
**WBC ($x10^9$/L)**	5.6 (1.4)	6.5 (1.9)	6.3 (1.9)
**Haemoglobin (g/dL)**	14.5 (1.4)	14.5 (11.7)	14.5 (10.7)
**Platelets ($x10^9$/L)**	241.3 (65.9)	250.9 (70.9)	249.4 (70.1)
**AST (U/L)**	26.4 (9.6)	24.7 (14.7)	25.0 (14.0)
**ALT (U/L)**	24.8 (12.2)	25.3 (16.8)	25.2 (16.1)
**ALP (U/L)**	65.9 (14.6)	68.1 (23.1)	67.8 (22.0)
**GGT (U/L)**	32.2 (21.3)	35.5 (58.5)	35.0 (54.5)
**Albumin (g/dL)**	4.5 (0.2)	6.8 (31.4)	6.5 (28.8)
**BUN (mg/dL)**	15.0 (3.8)	21.9 (13.4)	20.8 (12.6)
**Glucose (mg/dL)**	92.7 (5.8)	144.7 (57.4)	136.7 (56.1)
**HbA1c (%)**	5.5 (0.3)	7.9 (1.5)	7.5 (1.6)
**Insulin (μU/mL)**	8.4 (3.8)	16.5 (28.3)	15.4 (26.5)
**hsCRP (mg/dL)**	0.1 (0.1)	0.3 (1.3)	0.3 (1.2)
**Uric acid (mg/dL)**	5.4 (1.5)	6.0 (3.4)	5.9 (3.2)
**Total cholesterol (mg/dL)**	195.4 (29.3)	165.4 (36.9)	170.0 (37.3)
**LDL cholesterol (mg/dL)**	111.8 (30.2)	89.2 (30.0)	92.7 (31.1)
**HDL cholesterol (mg/dL)**	58.4 (21.2)	46.4 (13.8)	48.3 (15.7)
**Triglyceride (mg/dL)**	146.8 (91.7)	157.2 (98.2)	155.6 (97.1)
**UACR (mg/g creatinine)**	9.2 (13.8)	617.7 (1474.9)	569.7 (1377.1)
**Plasma AKR1B10 (pg/mL)**	283.1 (329.1)	667.5 (1215.2)	608.4 (1133.1)

Data are expressed as the mean (standard deviation) or n (%), unless otherwise specified. Abbreviations: SBP, systolic blood pressure; DBP, diastolic blood pressure; WBC, white blood cell; ALP, alkaline phosphatase; GGT, γ-glutamyl transpeptidase; BUN, blood urea nitrogen; HbA1c, haemoglobin A1c; hsCRP, high sensitivity C-reactive protein; LDL, low-density lipoprotein; HDL, high-density lipoprotein; UACR, urine albumin to creatinine.

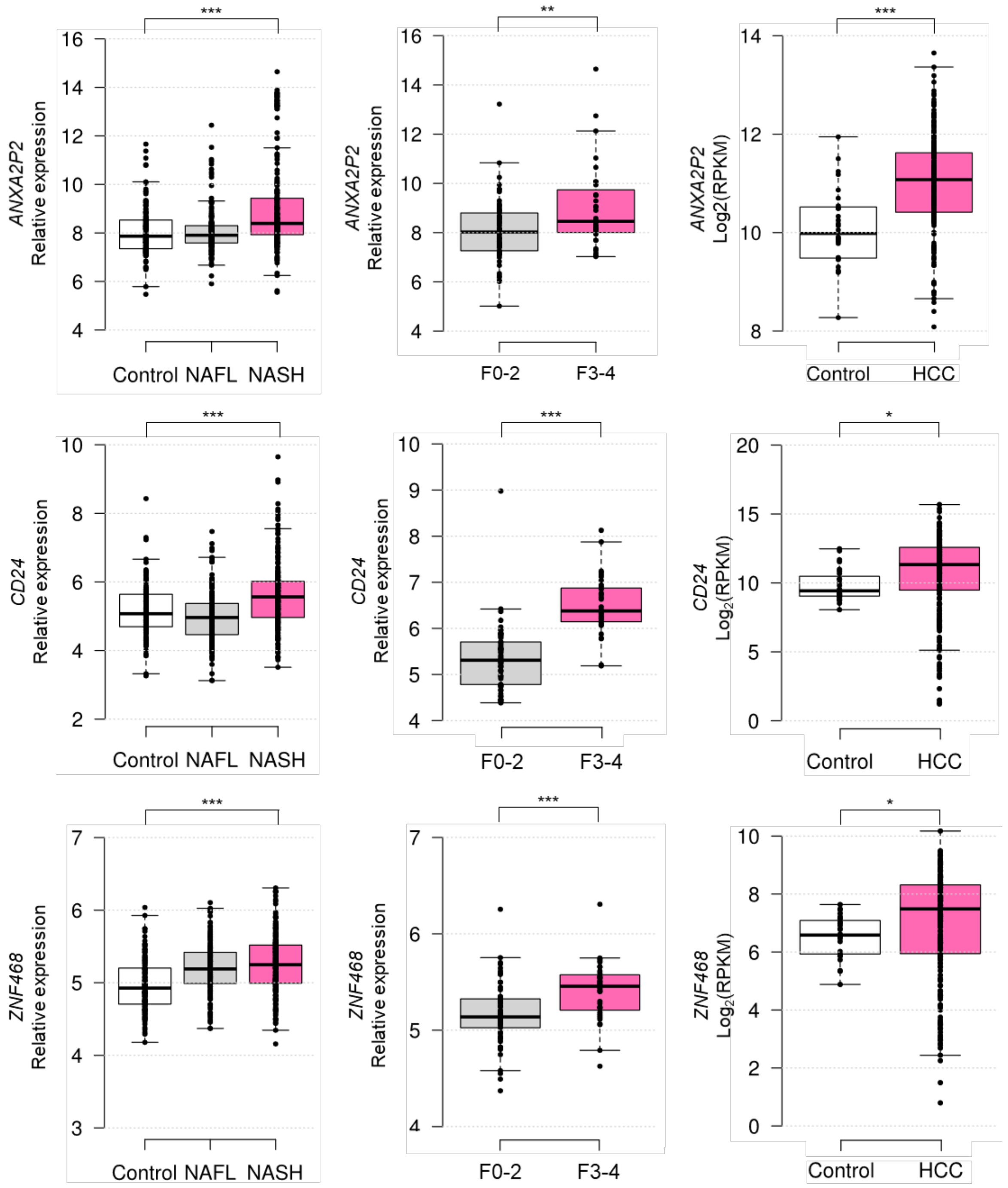

Supplementary Figure S1. Other putative secretory biomarker genes that showed a common stepwise upregulation according to NAFLD progression. *, $p < 0.05$; **, $p < 0.01$; and ***, $p < 0.001$.

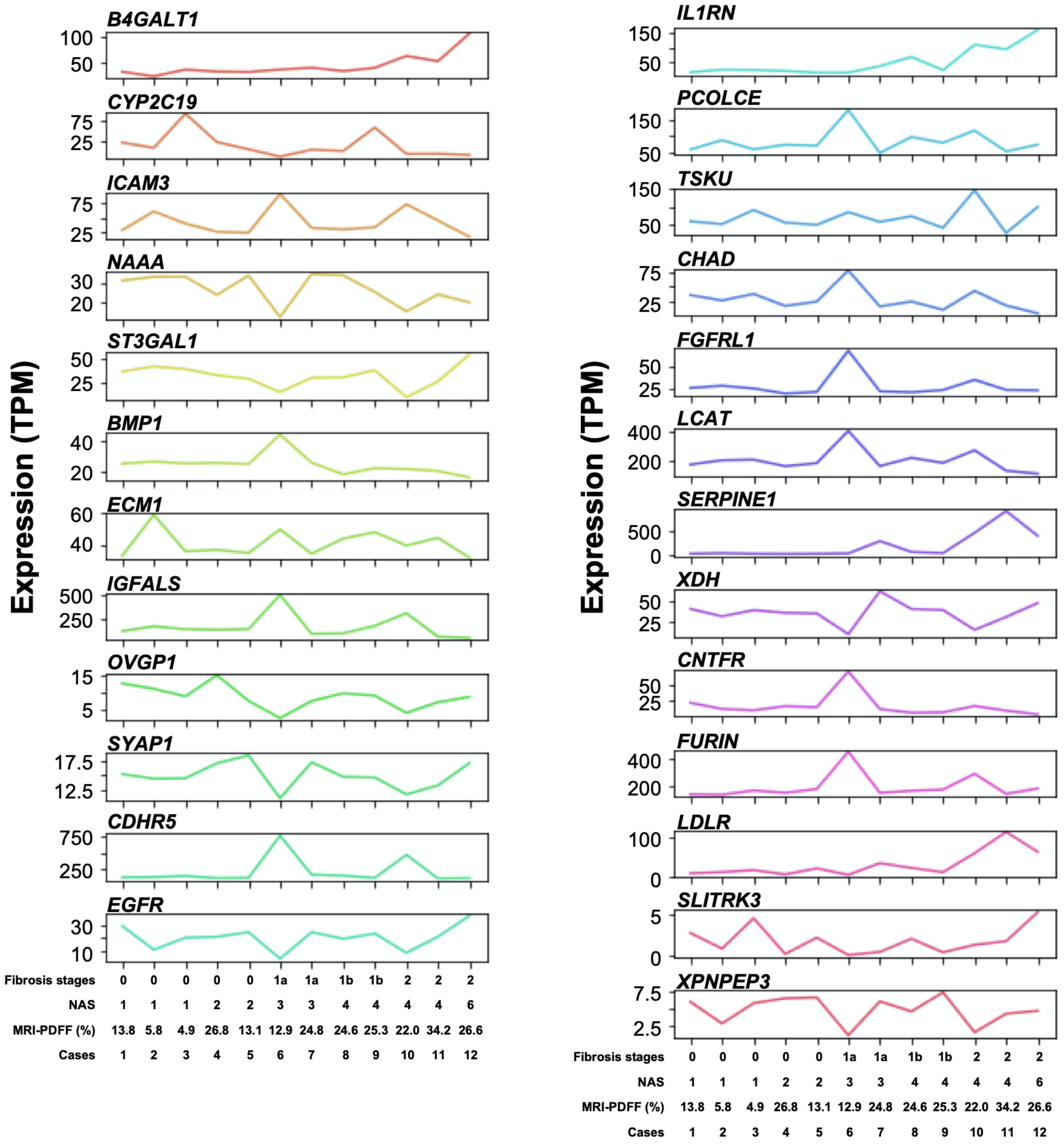

Supplementary Figure S2. RNA sequencing (RNA-Seq) data of 25 downregulated common differentially expressed genes listed in Figure 1B. RNA-Seq in our cohort was performed in liver samples from 12 study subjects with a spectrum of NAFLD progression, such as the NAFLD activity score (NAS) and fibrosis stage (F1-F4).

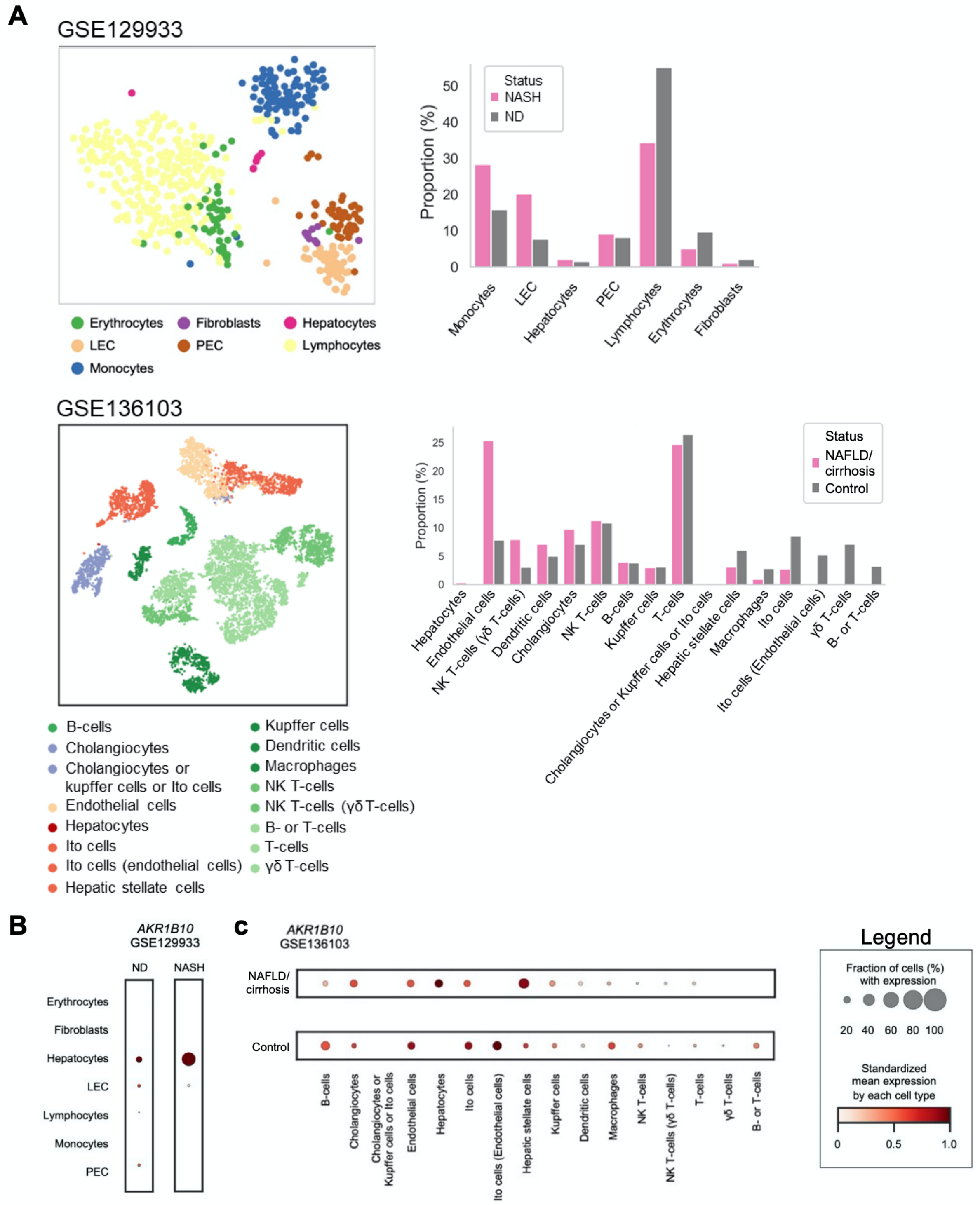

Supplementary Figure S3. The expression of AKR1B10 at the single cell level: Analyses of public single-cell RNA sequencing (scRNA-Seq) datasets. (**A**) Transitions in the cell type clustering and cell type proportion in scRNA-Seq data (GSE129933) of hepatic nonparenchymal cells from nondiseased control subjects (ND) vs. patients with NASH and in scRNA-Seq data (GSE136103) of hepatic CD45(-) cells from healthy control subjects vs. patients with NAFLD/cirrhosis from GEO. More information is provided in Supplementary Table S2. (**B** and **C**) Comparisons of *AKR1B10* expression in scRNA-Seq data (GSE129933) (**B**) of hepatic nonparenchymal cells from nondiseased control subjects vs. patients with NASH; and in scRNA-Seq data (GSE136103) (**C**) of hepatic CD45(-) cells from healthy

control subjects vs. patients with NAFLD/cirrhosis are depicted. Scale information about the fraction of cells that express AKR1B10 and the level of standardized mean expression by each cell type are presented in the legend box.

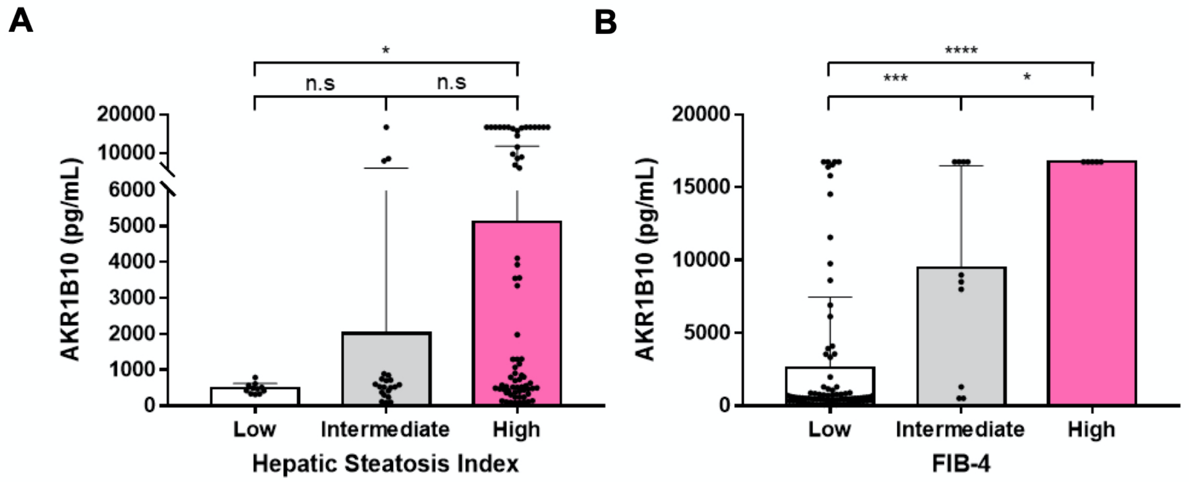

Supplementary Figure S4. Relationship between plasma AKR1B10 and NAFLD-related clinical scores in the replication study (*n*=102). Plasma AKR1B10 levels according to the likelihood of hepatic steatosis and advanced fibrosis based on the HSI (**A**) and FIB-4 (**B**) score systems (*n*=102). *, $p < 0.05$; ***, $p < 0.001$; ****, $p < 0.0001$; and n.s., not significant.

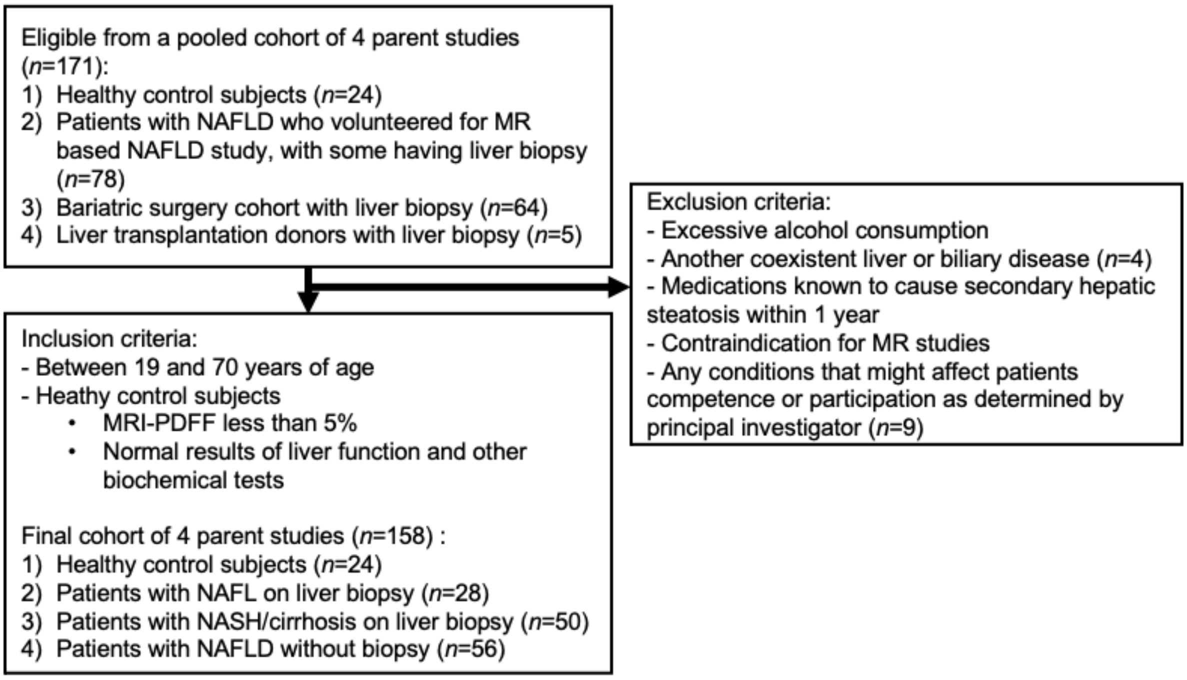

Supplementary Figure S5. Flowchart showing enrollment of study subjects in the replication cohort study.

Supplemental References

1. Traag, V.A.; Waltman, L.; van Eck, N.J. From Louvain to Leiden: guaranteeing well-connected communities. *Scientific Reports* **2019**, *9*, 5233, doi:10.1038/s41598-019-41695-z.
2. Soneson, C.; Robinson, M.D. Bias, robustness and scalability in single-cell differential expression analysis. *Nature Methods* **2018**, *15*, 255-261, doi:10.1038/nmeth.4612.
3. Uhlén, M.; Fagerberg, L.; Hallström, B.M.; Lindskog, C.; Oksvold, P.; Mardinoglu, A.; Sivertsson, Å.; Kampf, C.; Sjöstedt, E.; Asplund, A.; et al. Tissue-based map of the human proteome. *Science* **2015**, *347*, 1260419, doi:doi:10.1126/science.1260419.
4. Klopfenstein, B.J.; Kim, M.S.; Krisky, C.M.; Szumowski, J.; Rooney, W.D.; Purnell, J.Q. Comparison of 3 T MRI and CT for the measurement of visceral and subcutaneous adipose tissue in humans. *The British journal of radiology* **2012**, *85*, e826-830, doi:10.1259/bjr/57987644.
5. Bolger, A.M.; Lohse, M.; Usadel, B. Trimmomatic: a flexible trimmer for Illumina sequence data. *Bioinformatics* **2014**, *30*, 2114-2120, doi:10.1093/bioinformatics/btu170.
6. Kim, D.; Paggi, J.M.; Park, C.; Bennett, C.; Salzberg, S.L. Graph-based genome alignment and genotyping with HISAT2 and HISAT-genotype. *Nature Biotechnology* **2019**, *37*, 907-915, doi:10.1038/s41587-019-0201-4.
7. Babarinde, I.A.; Li, Y.; Hutchins, A.P. Computational Methods for Mapping, Assembly and Quantification for Coding and Non-coding Transcripts. *Comput Struct Biotechnol J* **2019**, *17*, 628-637, doi:10.1016/j.csbj.2019.04.012.
8. Kim, T.H.; Lee, K.; Park, I.B.; Choi, C.S.; Ahn, T.H.; Lee, D.H. The effects of DPP4 inhibitors on the levels of plasma catecholamines and their metabolites in patients with type 2 diabetes. *Diabetes research and clinical practice* **2019**, *156*, 107832, doi:10.1016/j.diabres.2019.107832.
9. López-Vicario, C.; González-Périz, A.; Rius, B.; Morán-Salvador, E.; García-Alonso, V.; Lozano, J.J.; Bataller, R.; Cofán, M.; Kang, J.X.; Arroyo, V.; et al. Molecular interplay between Δ5/Δ6 desaturases and long-chain fatty acids in the pathogenesis of non-alcoholic steatohepatitis. *Gut* **2014**, *63*, 344-355, doi:10.1136/gutjnl-2012-303179.
10. Ahrens, M.; Ammerpohl, O.; von Schönfels, W.; Kolarova, J.; Bens, S.; Itzel, T.; Teufel, A.; Herrmann, A.; Brosch, M.; Hinrichsen, H.; et al. DNA methylation analysis in nonalcoholic fatty liver disease suggests distinct disease-specific and remodeling signatures after bariatric surgery. *Cell metabolism* **2013**, *18*, 296-302, doi:10.1016/j.cmet.2013.07.004.
11. du Plessis, J.; van Pelt, J.; Korf, H.; Mathieu, C.; van der Schueren, B.; Lannoo, M.; Oyen, T.; Topal, B.; Fetter, G.; Nayler, S.; et al. Association of Adipose Tissue Inflammation With Histologic Severity of Nonalcoholic Fatty Liver Disease. *Gastroenterology* **2015**, *149*, 635-648.e614, doi:10.1053/j.gastro.2015.05.044.
12. Horvath, S.; Erhart, W.; Brosch, M.; Ammerpohl, O.; von Schönfels, W.; Ahrens, M.; Heits, N.; Bell, J.T.; Tsai, P.C.; Spector, T.D.; et al. Obesity accelerates epigenetic aging of human liver. *Proc Natl Acad Sci U S A* **2014**, *111*, 15538-15543, doi:10.1073/pnas.1412759111.
13. Frades, I.; Andreasson, E.; Mato, J.M.; Alexandersson, E.; Matthiesen, R.; Martínez-Chantar, M.L. Integrative Genomic Signatures Of Hepatocellular Carcinoma Derived

from Nonalcoholic Fatty Liver Disease. *PLOS ONE* **2015**, *10*, e0124544, doi:10.1371/journal.pone.0124544.

14. Xanthakos, S.A.; Jenkins, T.M.; Kleiner, D.E.; Boyce, T.W.; Mourya, R.; Karns, R.; Brandt, M.L.; Harmon, C.M.; Helmrath, M.A.; Michalsky, M.P.; et al. High Prevalence of Nonalcoholic Fatty Liver Disease in Adolescents Undergoing Bariatric Surgery. *Gastroenterology* **2015**, *149*, 623-634.e628, doi:10.1053/j.gastro.2015.05.039.
15. Lefebvre, P.; Lalloyer, F.; Baugé, E.; Pawlak, M.; Gheeraert, C.; Dehondt, H.; Vanhoutte, J.; Woitrain, E.; Hennuyer, N.; Mazuy, C.; et al. Interspecies NASH disease activity whole-genome profiling identifies a fibrogenic role of PPARα-regulated dermatopontin. *JCI Insight* **2017**, *2*, doi:10.1172/jci.insight.92264.
16. Haas, J.T.; Vonghia, L.; Mogilenko, D.A.; Verrijken, A.; Molendi-Coste, O.; Fleury, S.; Deprince, A.; Nikitin, A.; Woitrain, E.; Ducrocq-Geoffroy, L.; et al. Transcriptional network analysis implicates altered hepatic immune function in NASH development and resolution. *Nature Metabolism* **2019**, *1*, 604-614, doi:10.1038/s42255-019-0076-1.
17. Murphy, S.K.; Yang, H.; Moylan, C.A.; Pang, H.; Dellinger, A.; Abdelmalek, M.F.; Garrett, M.E.; Ashley-Koch, A.; Suzuki, A.; Tillmann, H.L.; et al. Relationship between methylome and transcriptome in patients with nonalcoholic fatty liver disease. *Gastroenterology* **2013**, *145*, 1076-1087, doi:10.1053/j.gastro.2013.07.047.
18. Tamburini, B.A.J.; Finlon, J.M.; Gillen, A.E.; Kriss, M.S.; Riemondy, K.A.; Fu, R.; Schuyler, R.P.; Hesselberth, J.R.; Rosen, H.R.; Burchill, M.A. Chronic Liver Disease in Humans Causes Expansion and Differentiation of Liver Lymphatic Endothelial Cells. *Front Immunol* **2019**, *10*, 1036, doi:10.3389/fimmu.2019.01036.
19. Ramachandran, P.; Dobie, R.; Wilson-Kanamori, J.R.; Dora, E.F.; Henderson, B.E.P.; Luu, N.T.; Portman, J.R.; Matchett, K.P.; Brice, M.; Marwick, J.A.; et al. Resolving the fibrotic niche of human liver cirrhosis at single-cell level. *Nature* **2019**, *575*, 512-518, doi:10.1038/s41586-019-1631-3.
